# Quality Evaluation of Fresh Pasta Fortified with Sourdough Containing Wheat Germ and Wholemeal Semolina

**DOI:** 10.3390/foods12142641

**Published:** 2023-07-08

**Authors:** Pasquale Catzeddu, Simonetta Fois, Valentina Tolu, Manuela Sanna, Angela Braca, Ilaria Vitangeli, Roberto Anedda, Tonina Roggio

**Affiliations:** Porto Conte Ricerche Srl, Località Tramariglio, 07041 Alghero, Italy; fois@portocontericerche.it (S.F.); tolu@portocontericerche.it (V.T.); sanna@portocontericerche.it (M.S.); braca@portocontericerche.it (A.B.); vitangeli@portocontericerche.it (I.V.); anedda@portocontericerche.it (R.A.); roggio@portocontericerche.it (T.R.)

**Keywords:** durum wheat, vitamin E, nuclear magnetic resonance, lipids, antioxidants

## Abstract

Pasta is a staple food in the Mediterranean diet, primarily manufactured with two essential ingredients, semolina and water; nowadays, it is often supplemented with functional ingredients. In this work, a sourdough obtained with wheat germ and wholemeal semolina was used, in order to improve sensorial and nutritional properties of fresh pasta, to prevent lipids oxidation, and to improve the shelf life. Three different formulations were prepared, a first one using semolina, a second one with raw wheat germ, wholemeal semolina, and semolina, and the last one with semolina and sourdough. The study highlighted the improved nutritional properties of pasta with sourdough (reduced phytic acid content, higher antioxidant activity and phenolic content). Proteins, ashes, dietary fibers, lipids, and tocols (vitamin E) increased in pasta with wheat germ and wholemeal semolina, and with sourdough. The amount of tocols decreased in pasta samples after cooking, except for the β–tocopherol in sourdough pasta, the amount of which remained high, surprisingly. Lipase and lipoxygenase enzymes likely decreased as an effect of the pasteurization process. The NMR analysis showed that lipid oxidation was higher in semolina pasta than in pasta with wheat germ, most likely due to the protective effect of antioxidants deriving from wheat germ.

## 1. Introduction

Wheat kernel is constituted by three main fractions, which are the central endosperm (80–85%), the bran layers (aleurone and pericarp; 13–17%), and the germ or embryo (2–3%) [1]. During dry milling, the endosperm is turned into white flour or semolina, depending on the species of *Triticum*, whereas bran and germ are separated and represent a by-product of the milling process, mostly used as livestock feed.

Despite having been historically identified as by-products of the milling industry, both germ and bran are being increasingly considered valuable sources of nutrients [2,3]. Wheat germ contains carbohydrates (51%) with dietary fibers accounting for the 12%, proteins (28%), lipids (10%), and a wide variety of functional bioactive compounds, such as tocopherols, carotenoids, vitamins, phytosterols, phytochemicals (e.g., polyphenols), and minerals. Albumins and globulins, the most represented proteins, guarantee a well-balanced amino acid profile. Bran is an excellent source of dietary fiber, and is also rich in minerals, vitamins, and some phytochemicals with antioxidant capacity, such as polyphenols and alkylresorcinols [3]. 

Taking into account their high nutritional value, many studies pointed out the positive physiological effects of such by-products. The consumption of bran fibers was proved to give health benefits, reducing the risk of gastrointestinal and cardiovascular diseases, colon and breast cancers, and obesity [4]. The high lipid content of wheat germ, mainly composed of unsaturated fatty acids (UFA) such as oleic, linoleic, and α-linolenic acids, suggests that germ can be used as a dietary supplement in human diet. Hu et al. [5] demonstrated that whole grain intake, including wheat germ, significantly reduces coronary heart disease risk. Marangoni et al. [6] reported a reduced incidence of cardiovascular diseases associated with the consumption of linoleic acid, which is the most abundant fatty acid in wheat germ. Ghafoor et al. [7] described, in a review article, the functional properties of wheat germ oil, obtained by mechanical pressing or solvents extraction, and its utilization in medicine, cosmetic industry, food production, and biological insect control. Particular attention was given to the beneficial effects of oil constituents, in particular fatty acids and vitamin E.

Along with the positive elements, the germ and bran are known to contain some anti-nutritional factors, as agglutinin, a protein involved in gluten intolerance, and phytic acid, which complexes the minerals reducing their bioavailability. The presence of agglutinins has been reported in wholemeal pasta enriched with wheat germ, but it disappeared after pasta cooking [8]. Another negative aspect is the absorption of pesticides used in agricultural crops [1], being that germ and bran are allocated in the outer part of the kernel and pesticides are highly attracted by fats. Among the use of germ in food production, the large amount of lipids and the presence of lipase and lipoxygenase enzymes make the incorporation of wheat germ into flours undesirable. Actually, the hydrolytic and oxidative enzymatic activity is the main trigger of the rapid development of rancidity, which is considered detrimental for the shelf life and for the quality of flours and bakery products. The use of bran in food production can also be considered disadvantageous; in bread-making, it prevents a continuous gluten-matrix development and lowers the loaf volume; in bread and pasta, inclusion of bran gives rise to unpleasant sensory properties, such as a strawy or sandy mouth feeling as well as unpleasant bitter and astringent taste and aftertaste. Such undesirable effects, linked to the use of ingredients containing phenolic compounds, fatty acids, and small peptides [9,10], discourage producers from adding bran or germ to food formulations and consumers from increasing the daily intake of wholemeal pasta or bread, as observed by Laureati et al. [11]. 

The potential of microbial fermentation to improve the nutritional, textural, and sensory quality of food has been largely investigated in cereal flours and cereal by-products [12,13,14]. Fermentation of bran and germ was found to reduce some anti-nutritional factors, such as agglutinin and phytic acid, and to decrease the enzymatic activity of lipase and lipoxygenase, which may concur to the development of rancid flavor in non-fermented, germ-containing flours [15,16]. The use of sourdough-fermented wheat germ in bread-making was found to give an overall improvement of nutritional, texture, and sensory properties [17]. 

In this study, a sourdough obtained from spontaneously fermented wheat germ and wholemeal semolina was used to make fresh pasta in combination with semolina. A second type of pasta was prepared using unfermented wheat germ, wholemeal semolina, and semolina. The control sample was prepared using exclusively semolina. Chemical-physical and cooking properties, together with nutritional and sensory characteristics of pasteurized fresh pasta samples, were determined. Furthermore, Nuclear Magnetic Resonance (1H-NMR) analysis, gas chromatographic analysis of fatty acids methyl esters (FAME), and microbiological and sensory analyses were carried out as a function of storage in order to study the evolution of lipids along time.

## 2. Materials and Methods

### 2.1. Raw Materials and Reagents

Wholemeal semolina (without germ), semolina, and durum wheat germ were purchased from Mulino Galleu (Ozieri, Italy). The wheat germ was grinded using the homogenizer Bimby^®^ TM6 (Vorwerk Italia s.a.s., Milano, Italy) at 10,200 rpm for 30 s, to obtain a particle size lower than 850 μm, then it was vacuum-packaged and frozen at −40 °C. The pH, total titratable acidity (TTA), moisture, ashes, protein, and gluten content of the raw materials were reported in Table S1. 

All analytical-grade reagents were purchased from Sigma-Aldrich S.r.l. (Milano, Italy), unless otherwise mentioned. All culture media were purchased from Oxoid (Basingstoke, Hampshire, UK).

### 2.2. Physicochemical Composition of Raw Materials and Samples 

Gluten content was determined on semolina and wholemeal semolina, according to the Approved Method 38-12.02, of the American Association of Cereal Chemists (AACC) [18]. 

Moisture and ashes were determined at 130 °C and at 580 °C, respectively, using the Thermogravimetric Analyzer Thermostep (Eltra GmbH, Haan, Germany) until weight stability onset. 

Protein content (Nx5.7), total titratable acidity (TTA), and pH were determined according to Fois et al. [19]. 

Lipid content was determined following the Folch method for lipid extraction [20] with some optimizations. Briefly, fresh pasta and wheat germ were pulverized in liquid nitrogen using a cryogenic mill (Spex SamplePrep 6875, Metuchen, NJ, USA), while semolina and wholemeal semolina were extracted as received. Samples were kept at −80 °C until extraction. About 10 g of accurately weighed frozen samples were subjected to lipid extraction by means of a two-step process, and total lipids were quantified. First, the sample was dissolved in 200 mL of a CHCl_3_:CH_3_OH 2:1 solution, then 60 mL of 0.9% NaCl solution was added to induce separation into two phases. Solid suspensions were subjected to several washing steps on a Buchner funnel with an overall 40 mL of the same CHCl_3_:CH_3_OH 2:1 solution. The obtained filtrate was vortexed for 1 min and centrifuged (Avanti centrifuge J-26 XPI, Beckman Coulter srl, Cassina de’ Pecchi, Italy) at 1000× *g* for 5 min at 4 °C. Of the two-phase system resulting from the Folch extraction, the chloroformic phase was collected from the bottom of the centrifuge tube and dried in a rotary evaporator system (Buchi R-210 with vacuum controller V-850 and membrane vacuum pump V-700 and heating bath B-491 fixed at 45 °C), and then drying was completed to constant weight over of a nitrogen gas flow. 

The color was determined on raw pasta according Fois et al. [19]. Color changes (ΔE) were determined in pasta samples, according to Equation (1):

(1) $$\Delta E=\sqrt{{\left(L_{2}^{*}-L_{1}^{*}\right)}^{2}+{\left(a_{2}^{*}-a_{1}^{*}\right)}^{2}+{\left(b_{2}^{*}-b_{1}^{*}\right)}^{2}}$$

0 < ΔE < 1—the difference is unnoticeable
1 < ΔE < 2—the difference is only noticed by an experienced observer
2 < ΔE < 3.5—the difference is also noticed by an unexperienced observer
3.5 < ΔE < 5—the difference is clearly noticeable
5 < ΔE—gives the impression that these are two different color
where L*_1_, a*_1_, b*_1_ refer to the color parameters of the control sample, and L*_2_, a*_2_, b*_2_ refer to the color parameters of the experimental sample [21].

### 2.3. Sourdough Preparation, Refreshment and Analysis

Sourdough was obtained from a blend of durum wheat germ (155 g) and wholemeal semolina (1000 g), hereafter referred to as GWS, mixed with an equal amount of water (*w*/*w*), acidified at pH 5.0 using lactic acid 90%, and spontaneously fermented by the native microorganisms of the raw materials. Fermentation was carried out at 25 °C for 8 h using the bioreactor AFTL5 (SITEP S.r.l., Voghiera, Italy). A daily back-slopping was done, from Monday to Friday, using GWS, mother sourdough, and sterile water, at a ratio of 1:1:1 (dough yield 200). After each fermentation step and through the weekend, the sourdough was kept at low temperature (5 ± 1 °C). The fermentation time was reduced at 6 h after the 5th back-slopping to avoid excessive acidification. Starting from the 22nd back-slopping, the fermentation time was lowered to 5 h and then the sourdough was used for pasta preparation. TTA and pH were monitored in refreshed sourdough. The lactic acid bacteria (LAB) and yeast microflora was monitored according to Fois et al. [22]. The average number of LAB and yeasts in sourdough was about 10^9^ cfu/g and 10^6^ cfu/g, respectively; the pH was about 4.2 and TTA was 15.5 mL (NaOH N/10). Before pasta preparation, about eight colonies of bacteria and yeast were picked up from MRS and Rose-Bengal agar plates, of the highest dilution, and re-streaked in fresh agar medium for purification. Purified strains of LAB and yeast were identified using the instrument MALDI Biotyper (MicroFlex™, Bruker Daltonik GmbH, Bremen, Germany), the software MBT Compass^®^ 4.1, and the attached libraries (Bruker Daltonik GmbH, Bremen, Germany). 

### 2.4. Pasta Making and Analysis

**Figure 1:**
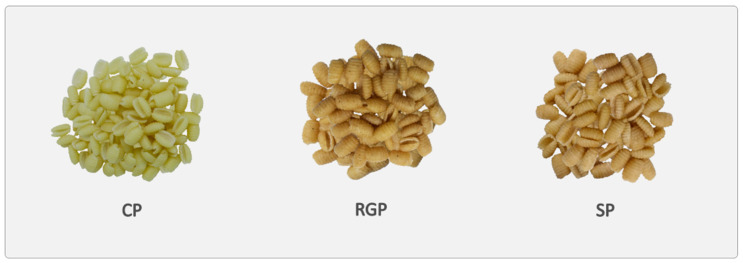
Raw pasta samples. CP = Control Pasta; RGP = Raw Germ Pasta; SP = Sourdough Pasta.

Three different formulations of fresh pasta (gnocchetto type; Figure 1) were prepared using the pasta maker La Monferrina Dolly (La Monferrina, Moncalieri, Italy), equipped with a bronze die:

1. CP (Control Pasta). Pasta made with semolina (1000 g) and water (300 g);
2. RGP (Raw Germ Pasta). Pasta made with 300 g of unfermented GWS (which contained 40 g of wheat germ and 260 g of wholemeal semolina), 700 g of semolina, and 300 g of water;
3. SP (Sourdough Pasta). Pasta made with 600 g of sourdough and 1000 g of semolina. The water (300 mL) was into the sourdough.

The amount of wheat germ in pasta mimicked the one in the kernel, which is about 3%. Three different replicates of each formulation were made, in three different days, using three different batches of semolina and whole meal semolina. The same batch of wheat germ was used. After production, fresh pasta was immediately pasteurized and packaged under modified atmosphere (MAP; CO_2_:N_2_ = 30:70), as in Fois et al. [22], then stored at 5 °C until analysis. All analyses were performed on the three formulations and the three replications (*n* = 9).

Total aerobic bacteria, LAB, and yeast were determined on pasta after pasteurization and after 1, 2, and 3 months of storage, according to Fois et al. [22]. Sensorial analysis was performed on cooked pasta samples at time zero, and after 1 and 2 months of storage. Raw and cooked pasta were homogenized with a cryogenic mill (SpexSamplePrep, Stanmore, UK) and stored at −30 °C before analysis. Total dietary fiber, protein digestibility, total phenolic compounds, antioxidant activity, and phytic acid were determined on cooked pasta. Lipase and lipoxygenase activity were determined on raw pasta. Vitamin E was determined on raw and cooked pasta. Lipid oxidation and FAME composition analyses were performed on lipid extract of raw pasta at time zero and after 15, 35, 55, and 75 days of storage.

### 2.5. Cooking Quality and Texture Analysis of Pasta

The optimum cooking time (OCT) and the cooking loss (CL) of pasta were evaluated according to the AACC Approved Method 66-50.01 [18]. The swelling index (SI) and the water absorption index (WAI) were calculated according to Padalino et al. [23]. SI indicated the grams of water in cooked pasta per grams of dry pasta. WAI evaluated the increase in pasta weight during cooking and was calculated as follows: (weight of cooked pasta–weight of raw pasta)/weight of raw pasta × 100.

Texture profile analysis (TPA) [24] was perfomed on cooked pasta using a TA.XTPlus Texture Analyser (Stable Microsystems, Godalming, UK), equipped with a 30 kg load cell. Pasta was cooked at OCT and immediately rinsed in cool water (4 °C) for 30 s to avoid overcooking. A single gnochetto was submitted to a double compression cycle using the pasta firmness-stickiness rig apparatus. Pre-test, test, and post-test speed were 2, 1, and 5 mm/s, respectively, and compression was set at 70%. Trigger force was 5 g and the wait time between first and second compression cycle was 5 sec. The software Exponent Stable Micro Systems (v6.1.16) was used for data processing and for calculation of hardness, adhesiveness, springiness, cohesiveness, gumminess, and chewiness. The reported TPA values are the average of 20 different determinations.

### 2.6. Nutritional Properties 

#### 2.6.1. In Vitro Protein Digestibility

Protein digestibility was determined on freeze-dried cooked pasta samples, according to the method of Pasini et al. [25] with minor modifications. Pasta sample (240 mg) was suspended in 16 mL of 0.02 N HCl (pH 2.0) containing 0.05 mg/mL of pepsin (≥3.200 units/mg, pepsin from porcine gastric mucosa, Sigma-Aldrich, Milano, Italy), and incubated at 37 °C in a shaking water bath for 30 min. Pepsin/protein ratio was 1:30 (*w*/*w*). Afterwards, a solution of 4.6 mL of 1 M boric acid and 0.5 N NaOH, adjusted to pH 6.8 with 5 N HCl, containing 0.25 mg/mL of pancreatin (8 × USP specifications, pancreatin from porcine pancreas, Sigma-Aldrich), was added. Pancreatin/protein ratio was 1:21 (*w*/*w*). The reaction was left to proceed for an additional 150 min in the shaking water bath at 37 °C, and finally it was stopped by adding 1 volume of trichloroacetic acid (20%, *w*/*v*). Samples after reaction were left overnight at 9 °C and then centrifuged (8000× *g*, 10 min, 24 °C). The resulting pellet was lyophilized and analyzed for nitrogen content by the AACC combustion method 46-30 [18], using a Rapid N Cube analyzer (Elementar Analysensysteme GmbH, Langenselbold, Germany). Total protein content was also determined on undigested samples, and the digestibility and protein availability were calculated [26]. The digestibility refers to the quantity of proteins digested over total protein content in cooked pasta, and it was calculated as follow: [(total protein content—protein content after digestion) × 100/total protein content]. The protein availability refers to the quantity of digested proteins over pasta dry basis, and it was obtained with the following formula: [(protein digestibility × protein content in cooked pasta)/100]. 

#### 2.6.2. Phytic Acid and Total Dietary Fiber Determination

Phytic acid and total dietary fiber (TDF) content was determined on freeze-dried cooked pasta using the Phytic Acid (Phytate)/Total Phosphorus assay kit (Megazyme, Wicklow, Ireland) and the Total Dietary Fiber assay kit (Megazyme, Wicklow, Ireland), respectively.

#### 2.6.3. Total Phenolic Content and Antioxidant Activity

Total phenolic content (TPC) and DPPH (2,2-diphenyl-1-picrylhydrazyl) radical scavenging activity were determined on cooked pasta samples, suspending 1 g of homogenized sample in 10 mL of an 80% aqueous methanol solution (20:80, *v*/*v*). The mixture was shaken for 2 h at 750 rpm in a thermomixer (Thermomixer Comfort, Eppendorf srl, Milano, Italy), then centrifuged at 800× *g* for 10 min. Supernatant was filtered through a 0.22 µm PTFE syringe filter (Phenomenex, Macclesfield Cheshire, UK) and stored at −20 °C until analyses. 

TPC of sample extracts was determined following the Folin–Ciocalteau method [27] and the modifications reported in Fois et al. [19].

The antioxidant activity of pasta was determined according to Boroski et al. [28] and the modifications reported in Fois et al. [19], and it was measured as percentage of discoloration of DPPH solution of pasta sample with respect to a blank sample.

### 2.7. Lipase and Lipoxygenase Activity

Tributyrin (Sigma-Aldrich) was used as the substrate to measure the lipase activity in GWS (1 g), sourdough (2 g), and freeze-dried uncooked pasta samples (1 g). The analysis was performed in 15 mL of 25 mM TrisHCl (pH 7.0), added with 100 μL of tributyrin and 25 μL of Tween 20 (Riedel de Haen AG, Seelze, Germany), and emulsified with the Ultra-Turrax Homogenizer T25 (IKA^®^-Werke GmbH & Co. KG, Staufen, Germany), equipped with a 10 mm dispersing head (S25N-10G), for 30 s at 10,000 rpm; then, the sample was added and the pH was immediately adjusted to 7.0 using 1 M NaOH. The reaction mixture was left stirring at 37 °C for 2 h and subsequently the pH variation was corrected to 7.0 using 10 mM NaOH with the automatic titrator pH-Matic 23 (Crison, Alella, Spain). A blank test was conducted by measuring the variation in pH in the homogenate of tributyrin and Tween 20, and the volume of NaOH necessary to correct the pH to 7.0 was subtracted from the final volume of NaOH used for sample titration. The lipase activity was calculated as micromoles of NaOH consumed per gram per hour of sample dry bases. 

The Lipoxygenase (LOX) activity was measured in 1 g freeze-dried sample of GWS, sourdough, and uncooked pasta samples. LOX extraction and determination of enzymatic activity was performed according to the method described by Tolouie et al. [29]. Briefly, LOX enzyme was extracted by mixing 1 g of the sample with 10 mL of 0.1 M potassium phosphate buffer (pH 7.0). The mixture was stirred for 1 h at room temperature and then centrifuged at 9000× *g* for 15 min at 4 °C, followed by a filtration of the supernatant using a 0.22 µm filter PVDF membrane (Millex, Merck KGaA, Darmstadt, Germany). The filtered supernatant was used in the reaction assay as LOX extract.

The substrate solution was prepared as follows: 0.5 mL of Tween 20 was dissolved in 10 mL of 0.1 M borate buffer (pH 9.0) and 0.5 mL of linoleic acid (>99% Sigma) was added drop by drop. Then, 1.3 mL of 1 M NaOH was added to achieve a clear and transparent solution. Finally, 90 mL of borate buffer was added, and the final volume of 200 mL was obtained with distilled water.

The enzymatic activity of LOX extract was carried out at room temperature, mixing in a quartz cuvette 0.04 mL of substrate solution, 0.92 mL of 0.1 M potassium phosphate buffer (pH 6.0), and adding 0.04 mL of LOX extract. After 2 min of reaction, the absorbance was read at 234 nm using a Cary-60 spectrophotometer (Agilent Technologies, Santa Clara, CA, USA). The blank value, obtained before the addition of LOX extract, was subtracted from the value obtained after reaction. For each sample, a double extract was prepared, and the enzymatic activity was determined in quadruplicate for each extract. LOX activity was reported as micromoles of hydroperoxides formed per minute per gram of sample (dry bases), using a molar absorption coefficient of 2.5 × 10^4^ M^−1^ cm^−1^ [30].

### 2.8. Determination of Vitamin E by HPLC Analysis 

#### 2.8.1. Materials and Standard Solution Preparation

Rac-α-tocopherol (α-TP), rac-β-tocopherol (β-TP), α-tocotrienol (α-TT), and β-tocotrienol (β-TT) were obtained from Vinci-Biochem Srl (Florence, Italy). All solvents used were of chromatographic grade, purchased from Merck (Darmstadt, Germany). 

#### 2.8.2. HPLC Conditions

According to Siji [31], the chromatographic analysis was performed on a 1260 Infinity II liquid chromatography system (Agilent Technologies, USA) equipped with a UV detector of the same series, using an Infinity Lab Poroshell 120, EC-C18 column (4 mm, 4.6 × 100 mm) (Agilent Technologies, Santa Clara, USA). The elution was carried out in gradient mode using a binary solvent mixture composed of 95:5; water:tetrahydrofuran (THF) with 0.05% acetic acid (solvent A) and 75:25:5; Acetonitrile:Methanol:THF with 0.035% acetic acid (solvent B) under the following conditions: from 30% to 75% B, 0–3 min; from 75% to 100% B, 3–8 min; 100% B from 8 to 15 min; 30% B to complete 17 min run time. The injection volume was 20 μL, the flow rate was 1.0 mL/min, the wavelength for UV detection was 295 nm. Data collection and handling was carried out by OpenLAB CDS Software (Agilent Technologies, Santa Clara, CA, USA).

#### 2.8.3. Preparation of Standards Solutions

Each standard was precisely mixed to obtain a 1.0 mL standard mix of fat-soluble vitamins at different concentrations. Linearity levels were prepared by subsequent dilution of standard spike mix, using solvent B as diluent. The linearity standard solutions were covering a range from 0.5 to 150 ppm. Samples were transferred to labeled brown analysis vials and stored at −32 °C.

#### 2.8.4. Sample Extraction

Vitamin E extraction was carried out in freeze-dried raw and cooked pasta, semolina, wholemeal semolina, GWS, and sourdough, according to Nurit et al. [32] and Karakas et al. [33], with the following modifications: briefly, 0.4 g of the freeze-dried samples were weighed and moistened in test tubes containing 1 mL H_2_O, and then 1 mL of ethanol was added. After addition of 2 mL hexane, the samples were vortexed for 60 s and centrifuged at 1434× *g* for 5 min. For each sample, the extraction was repeated twice, and the collected hexane portions were combined and evaporated to dryness. The remaining residue was dissolved with 100 μL of solvent B, filtered through a 0.22 μm syringe filter, placed in labeled brown vials, and stored at −32 °C until HPLC analysis.

Recovery analyses for vitamin from the samples were performed by adding known amounts of vitamin standards (5 and 10 µg/mL) to the samples followed by the extraction procedure. The difference in detector response between the spiked and unspiked sample compared to the standard chromatogram was used to determine the vitamin content of the samples.

### 2.9. Lipid Oxidation Analysis

The expected development of rancidity was checked by studying lipid oxidation phenomena at molecular level. The most important involved species have been characterized on lipid extracts by means of ^1^H NMR analyses combined with the quantification of FAME using Gas-Chromatography (GC). Before NMR analysis, for each analytical sample, approximately 10 mg was accurately weighed and transferred into a 2 mL Eppendorf tube. Then, 900 μL of deuterated chloroform (CDCl3 99.8% D, Acros Organics, Geel, Belgium) with 0.03% (*v*/*v*) TMS, used as a chemical shift and quantitative reference standard, was added to the oil sample. Next, 800 μL of the NMR sample was transferred into 5 mm o.d. tubes for analysis.

All NMR spectra were acquired with a Bruker NMR Avance instrument (Bruker, Ettlinge, Germany) working at a frequency of 600 MHz. A BBI probe was used, and spectra were acquired at 298 ± 0.1 K (Bruker BVT3000, BCU05 temperature control system). NMR acquisition parameters were optimized according to preliminary analysis of the same system. In particular, the spectral window was optimized to observe all signals comprising aldehydes at low field (~9.8 ppm) (SW = 7000 Hz), while larger SW were acquired for selected samples to check for the presence of free fatty acids. The proton hard pulse (10.85 μs) was calculated for one representative sample and randomly checked on different samples throughout the work. Relaxation time was set to 5 s aiming at relative quantification of signals. Acquisition time was set as to allow complete collection of signal (AQ = 4 s). Overall, 64 scans were collected for each 1D ^1^H NMR spectra. Post processing of the free induction decay consisted in calibration, baseline correction, and apodization to a line broadening of 0.3 Hz. The softwares Bruker Topspin 2.1 and MNova 14.2.2 (MestreLab Research, Santiago de Compostela, Spain) were used for acquiring, processing, and visualizing NMR data.

### 2.10. GC Analysis of FAME 

Fatty acids compositions of pasta samples were assessed by GC analysis of FAME. FAME mixtures were obtained from lipid extract through a basic methylation protocol, following Siliani et al. [34]. Briefly, about 10 µL of lipid extract was dissolved in 1 mL of an n-hexane solution of internal standards (methyl valerate, methyl nonanoate, methyl tridecanoate, methyl nonadecanoate, 1 mg/mL each). Then, 40 µL of methanolic potassium hydroxide (2N) was added and the mixture vortexed for 1 min; the hexane phase was recovered for GC analysis. Methylations were carried out in duplicate for each lipid extract.

Methylated samples were analyzed following Melis et al. [35]. An Agilent 7890A gas chromatograph was used (Agilent Technologies, Wilmington, USA) equipped with an FID detector, split/splitless injection port (split mode, with split ratio of 50:1), an autosampler and a Supelco SP-2560 GC column (100 m × 0.25 mm internal diameter × 0.20 μm film thickness). GC temperature program was set to 45 °C (4 min), then to 175 °C (13 °C/min ramp, 27 min), and finally to 215 °C (4 °C/min ramp, 35 min). The system was controlled by the Agilent ChemStation (Version B.04.02). FAME identification was made by using standards purchased from Nu-Check Prep (Elysian, MN, STD #463, #674) and from Sigma-Aldrich (Merck KGaA, Darmstadt, Germany, 37-Component FAME Mix) and each FAME was expressed as a percentage of the total FAME.

### 2.11. Sensory Analysis

The study of consumer acceptability was performed on the three pasta samples just after packaging, and after 30 and 60 days of storage, by means of 52 regular pasta consumers (24 females and 28 males), aged between 28 and 62 years—inexperienced tasters. 

A written informed consent was obtained from all consumers before the sensory test and subjects with allergies or intolerances towards ingredients of pasta samples were excluded. All subjects were instructed to refrain from smoking and drinking (except water) before the sensory test. Pasta samples were provided to the consumer for a household analysis; they were asked to cook the pasta and to evaluate the following sensory properties: smell, appearance, taste, texture, and overall acceptability. A 9-point hedonic scale was used, ranging from 1, corresponding to “extremely disliked” (on the left side of the scale), to 9, corresponding to extremely liked (on the right side of the scale). Each consumer received 100 g of each sample, in a randomized and balanced order, packaged in modified atmosphere, and each package was marked with a three-digit code. The three pasta samples were provided within one week. Consumers were advised about the storage of pasta before consumption (low temperature, 5–8 °C), the optimal cooking time, and the seasoning after cooking. All sensory tests were performed after microbiological analysis of samples to ensure the suitability for human consumption, and facilities provided by Google (https://docs.google.com/forms/u/0/, accessed on 12 July 2022) were used. 

### 2.12. Statistical Analyses

Standard ANOVA procedure (randomized complete design with three replicates and three treatments) was applied on the data set. Means were separated by LSD test at *p* = 0.05 significance level, using Statgraphics Centurion software package (version 16.1.11, Statpoint Technologies Inc., Warrenton, VA, USA).

GC FAME data analysis was carried out using the web-based tool MetaboAnalyst 5.0 (Canada) (https://www.metaboanalyst.ca/MetaboAnalyst/ModuleView.xhtml, accessed on 5 October 2022). One-way ANOVA followed by Tukey’s test was performed (with no data preprocessing); both *p* < 0.05 and associated False Discovery Rate < 0.05 were used as significance threshold parameters. 

Sensory data analysis was performed using XLSTAT, version 2018.01 (Addinsoft, New York, NY, USA).

## 3. Results and Discussion

### 3.1. Physicochemical Characteristics of Pasta

The physicochemical properties of raw pasta are reported in Table 1. Moisture content was different in the three samples, being 24.09% in CP, 25.47% in RGP, and 26.32% in SP, due to the greater water retention capacity of the fiber in RGP and SP. The values comply with the limits established by the Italian law for fresh pasta (DPR no 187, 9 February 2001). The addition of GWS increased protein, ashes, and lipid content in both RGP and SP, as previously shown in other studies [36,37]. The acidity in SP was the highest, as expected, with lower pH and higher TTA values than CP and RGP. With respect to the acidity of CP and RGP, no significant differences were observed in pH, whereas TTA values were significantly different, likely due to the free fatty acids of wheat germ. 

Regarding the color, which is the first factor evaluated by consumers when purchasing pasta, GWS addition has a significant effect. In RGP and SP, the lightness (L*) and the yellow index (b*) are lower, and the red index (a*) is higher than in CP. The lower brightness and the higher red index might be the effect of the Maillard reaction between sugars and amino acids of wheat germ, induced by the pasteurization of pasta, as postulated by Pinarli et al. [36] and Boukida et al. [1]. The decrease in lightness and increase in redness was noticed by many other authors in foods added with wheat germ [21,36,37]. The addition of fermented GWS in SP increased the lightness (L*) and the red index (a*), compared to RGP containing unfermented GWS, and decreased the yellow index (b*). The overall color difference of RGP and SP against CP, represented by the ΔE value reported in Table 1, can be considered “clearly noticeable” for both, but the value was higher for RGP than SP.

### 3.2. Cooking Quality

Data in Table 2 show the cooking quality parameters of pasta. The optimal cooking time (OCT) decreased, and the cooking loss increased from CP to RGP and SP. Despite the reduction in the cooking time, which means less time in boiling water, RGP and SP showed a higher release of organic substances in the water. Among other studies on OCT, some authors [19,38] found the same OCT in fortified and not fortified fresh pasta, whereas a review paper reported a reduction in OCT after addition of non-durum wheat ingredients [39]. The increase in organic substances in cooking water can be explained by the effect of gluten dilution, or of bran and germ interference with the development of the gluten matrix and the gluten-starch interaction, which reduces the entrapment of swollen starch granules during boiling, thus leading to a greater cooking loss [39,40]. The cooking loss in SP was higher than RGP, most likely because the gluten matrix was further weakened by the proteolytic action of the fermentation process. The values of swelling index and water absorption index decreased from CP to RGP and SP, likely due to the shorter cooking time. Teterycz et al. [21] postulated a lower water absorption capacity of wheat bran and wheat germ proteins than gluten proteins.

### 3.3. Textural Properties

The results of TPA analysis on cooked pasta are reported in Table 3. All TPA parameters were lower in SP than in CP and RGP, whereas CP and RGP showed similar textural properties, except for springiness, that was lower in RGP (0.87) than in CP (0.91). In this study, the addition of GWS did not affect the texture of fresh pasta in comparison with the control pasta, despite the increase in lipids and protein content (Table 1). Several studies found that the addition of lipids affect the properties of pasta (higher firmness, lower stickiness, and cooking loss), due to the formation of lipid-amylose complexes and the decrease in starch solubility [41,42]. 

Other studies found an increase in firmness in pasta and bread prepared with wheat germ. In pasta, it was ascribed to the increased protein content [21], and in bread to the hindered gluten formation, most likely due to the glutathione added with the wheat germ [15]. On the contrary, Vignola et al. [43] found a weakening of pasta structure after fiber addition, likely due to the hindered formation of the gluten matrix. The reduction in the texture parameters in SP, except the adhesiveness, can be associated with the weakening of the gluten matrix in acidified substrates, due to gluten-starch interaction or the proteolytic activity of microorganisms [44]. Schettino et al. [45] reported that the use of fermented wheat germ and bran in dried pasta reduced the hardness but increased the chewiness and the cohesiveness.

### 3.4. Nutritional Properties 

Data on in vitro protein digestion, phytic acid content, total dietary fiber, total phenolic content, and antioxidant activity of cooked pasta samples were reported in Table 4. The content of Vitamin E in raw materials and pasta (raw and cooked) was reported in Table 5.

#### 3.4.1. In Vitro Protein Digestion Analysis

The results of in vitro digestion of pasta showed significant differences between samples: CP was the less digestible sample (49.58%) and RGP was the most digestible (57.67%). SP showed an intermediate digestibility value (54.10%). The protein availability followed the same trend of digestibility. Fois et al. [19] already observed a lower digestibility in fresh pasta fortified with sourdough, and this result was attributed to the pH-drop method used. The in vitro digestibility method used in this study measures the nitrogen content of undigested proteins, thus the reduced digestibility in SP could be ascribed to the fermentation process, which might have already hydrolyzed some proteins or have induced protein aggregation or formation of protein-starch complexes that reduced the proteases activity. The role of phenolic compounds on protein digestibility has also been suggested. Several authors hypothesized a decrease in in vitro protein digestibility due to the formation of complexes between proteins and phenolic compounds, which inhibited the activity of proteolytic enzymes [26,46]. Sze-Tao and Sathe [47] observed the formation of insoluble complexes after binding of walnut proteins with solubilized phenolic compounds. Furthermore, the accessibility of proteases to their sites of action is fundamental for digestibility. The more compact structure of CP, as shown by the highest values of TPA parameters, could give an explanation to the lowest digestibility value.

#### 3.4.2. Phytic Acid Content

The lowest amount of phytic acid was found in pasta with semolina (CP, 0.17 g/100 g dry basis (d.b.)) as expected, and the highest value in pasta with raw germ (RGP, 0.36 g/100 g d.b.). The fermentation of GWS lowered the phytic acid content, which was 0.18 g/100 g d.b. in the SP sample, according to a previous work describing the use of fermented whole-wheat semolina for pasta making [19]. Bran and germ are a good source of minerals (calcium, potassium, magnesium, etc.), but, unfortunately, they are also rich in phytic acid. The latter can chelate mineral cations, forming insoluble complexes and reducing their bioavailability in wholewheat-based foods. The sourdough acidification activates the phytase enzyme, endogenous of wheat, which dephosphorylates the phytic acid, thus reducing its chelating activity and increasing the bioavailability of minerals [48].

#### 3.4.3. Total Dietary Fiber

The amount of total dietary fibers was higher in RGP (8.34 g/100 g d.b.) and SP (8.66 g/100 g d.b.) than in CP (6.32 g/100 g d.b.), as expected, due to the use of GWS. The higher amount of total dietary fibers in SP than in RGP, although statistically significant, was quite low and can be related to the higher cooking loss in SP, which may have determined a concentration of the insoluble fiber, or to the increase in resistant starch due to the fermentation process [22].

#### 3.4.4. Total Phenolic Content and Antioxidant Activity

The values of TPC and the antioxidant activity increased from CP to RGP and SP, and the highest values were found in SP to confirm what already seen elsewhere [19]. The fermentation of GWS led to an increase in phenolic compounds and the relative antioxidant activity, and this can be due to the breakdown of the cell walls and the liberation of the antioxidant compounds, according to Hur et al. [49].

#### 3.4.5. Vitamin E Content

Vitamin E is a natural antioxidant that is present in wheat grains with four vitamers: two tocopherols (α- and β-) and two tocotrienols (α- and β-). The tocopherol content is on average sixteen times higher in germ than in bran, whereas tocotrienol content is twice higher in bran than in germ [50,51,52]. 

In this study, the analysis of raw materials (Table 5) revealed that semolina had the lowest values of total tocols (36.7 µg/g d.b.), GWS had the highest value (77.9 µg/g d.b.), and wholemeal semolina was in between (64.4 µg/g d.b.). This is in agreement with values found in literature, which reports that wheat germ is the richest fraction with an average content of 271 µg/g d.b. of tocols, followed by the bran fraction (95 µg/g) [53]. Apparently, fermentation did not affect the total content of tocols, which was 77.5 µg/g d.b. in sourdough (Table 5).

Analogous results were found in raw pasta (Table 5), where CP showed the lowest value of total tocols (22.1 µg/g d.b.), whereas RGP and SP showed almost the same amount of total tocols (54.5 and 55.9 µg/g d.b.)—about two times higher than CP. The decrease in tocols has been documented in sourdough preparation and dough-making, likely due to sensitivity to contact with air [12], and over the whole pasta-making process [54], which includes critical steps, such as an extensive mixing, the extrusion phase, and, finally, the thermal treatment in boiling water. After comparison of tocols content in raw and cooked pasta, a significant reduction in tocols was observed in all of the three samples (Table 5). Total tocols content was 27% lower in cooked CP sample compared to the raw CP sample, whereas RGP and SP samples lost about 65 and 35% of tocols, respectively, after cooking. The loss of tocols could have been due to their leaching into the cooking water, or to their decomposition, or reduction into another compound because of the hydrothermal treatment of cooking. To corroborate the first hypothesis, the greater cooking loss of tocols in RGP and SP than in CP (Table 5) could be explained by the physical presence of bran and germ, which probably interfere with the development of a continuous gluten matrix, as already found in our previous work [19].

In Table 5, the data of α and β tocopherols in raw and cooked pasta are shown. The analysis revealed that the content of α-tocopherol after cooking was dramatically reduced in RGP and SP samples (less 84.2% and 61.5%, respectively). Among tocols, the α-tocopherol is considered to have the highest antioxidant activity [7]. On the contrary the content of β-tocopherols after cooking decreased in RGP, whereas almost the same quantity of β-tocopherols was found in SP, indicating that, most likely, it remains completely entrapped in the food matrix during cooking. We did not find an explanation for such a phenomenon in the available literature but suggest that the conditions determined by fermentation (i.e., a lower pH, presence of acids and ethanol in the food matrix, hydrolysis of proteins and starch) could have been responsible for the development of new and strong interactions between β-tocopherols and the food matrix. Such interaction did not develop in the α-tocopherol, whose molecule differs from β-tocopherol (in the α-tocopherol the chromanol ring is completely methylated, whereas in the β- tocopherols, the ring contains two methyl groups).

### 3.5. Lipase and Lipoxygenase Activity 

The lipase and lipoxygenase activity were evaluated on GWS, sourdough, and pasta (d.b.). The results were reported as residual enzyme activity (Table 6) vs. the GWS value, considered as the reference value. The highest lipase activity was found in GWS, corresponding to 21.76 μmol NaOH per gram per hour. A lower value of the lipase activity was found in sourdough and, consequently, in SP, resulting in 31% and 18%, respectively. The samples CP and RGP did not show any lipase activity. As for lipase, the highest lipoxygenase activity was found in GWS, corresponding to 20.77 (μmol conjugated dienes per gram per minute), whereas in sourdough it was about 59% of the value registered in GWS. The lipoxygenase activity was lower in pasta samples, resulting in 20%, 27%, and 30% of the initial value, for CP, RGP, and SP, respectively. 

The lipase enzyme is responsible for the formation of free fatty acids, which leads to the rancidity appearance and the reduction in shelf life in flours and wheat-based products. The lipoxygenase is responsible for oxidative reactions and its activity is detrimental in pasta because it induces oxidative bleaching of yellow pigments and therefore the qualitative decay of pasta [55]. In this work, the highest values of lipase and lipoxygenase activity were found in GWS, as expected, because the enzymes are mainly concentrated in wheat bran and germ [56,57]. The activity of wheat germ lipase and lipoxygenase is known to be reduced by conventional heat treatments (dry and wet heating), microwave heating, or infrared treatment [1,58]. In this work, the absence of lipase in CP and RGP samples (Table 6) most likely indicated the effectiveness of pasteurization to inactivate the enzyme coming from the wheat kernel. The fermentation had a significant effect on the enzyme content: the content of lipase and lipoxygenase was 70% and 41% lower in S than GWS, respectively. Marti et al. [15] found a complete inactivation of lipases after wheat germ fermentation, whereas Rizzello et al. [59] found a reduction in lipase activity in fermented wheat germ, of about 2.6-fold compared to raw germ. Interestingly, in this work the lipase activity was still present in SP, even after pasteurization. We suggest that spontaneous sourdough microorganisms, which had their origin from wheat germ, produced a heat-resistant enzyme. In order to verify this hypothesis, the lipolytic activity of the strains isolated from sourdough were assessed using a plate assay test [60] and tributyrin as a substrate. The results showed that five yeast strains, belonging to the species *Kazachstania servazzii*, *Pichia fermentas,* and *Clavispora lusitaniae*, out of eight isolated from sourdough, were able to produce lipases, as evidenced by the halos around the colonies (Figure S1). The microorganisms isolated and identified are reported in Table S3. With regard to lipase-producing microorganisms, Syrokou et al. [60] isolated lipase-producing LAB from wheat sourdough, 11 strains over 207 tested, and Ciafardini et al. [61] isolated a lipase producing strains of *Saccharomyces cerevisiae* from olive oil. Note that the lipoxygenase activity measured in CP samples could be ascribed to germ and bran contamination of semolina, as reported by Hidalgo et al. [30].

### 3.6. Microbiological Analysis of Pasta

The microbiological analysis of pasta after pasteurization, reported in Table S2 in Supplementary Files, showed that LAB, yeast, and molds were not present or under the limit detection (10^2^ cfu/g) up to 3 months of storage. Total plate count analysis showed the presence of aerobic bacteria in the three samples, at all sampling times, but the values of cfu/g were quite low, approximately between 60 and 700 cfu/g. These data revealed the effectiveness of the pasteurization process in reducing the microflora of pasta, in spite of the high number of microorganisms introduced with raw ingredients and sourdough, as reported in Table S2. Moreover, the packaging at modified atmosphere and the cold storage prevented the development of aerobic microflora, regardless the ingredients used to make pasta.

### 3.7. Oxidative Degradation Products of Lipids 

**Figure 2:**
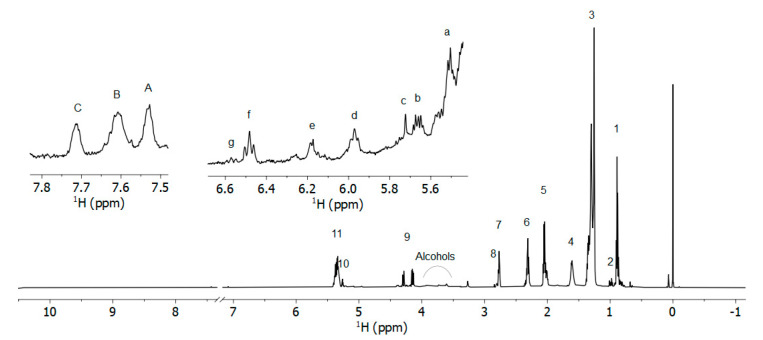
NMR spectrum of the lipid extract of a raw pasta sample. (1) Terminal −CH_3_ of SFA and ω−9, ω−7 and ω−6 MUFA; (2) Terminal −CH_3_ of ω−3 PUFA; (3) −(CH_2_)_n_− of all FA; (4) −OCO−CH_2_−C**H**_2_− of all FA except for ARA; (5) −C**H**_2_−CH=CH− in β position; (6) −OCO−C**H**_2_− in α position; (7) =HC−C**H**_2_−CH= of ω−6 DUFA; (8) =HC−C**H**_2_−CH= of triunsaturated FA; (9) sn−1,3 glyceryl protons; (10) sn−2 glyceryl protons; (11) −**H**C=C**H**− of all unsaturated FA. (a–g) signals from conjugated double bonds associated with hydroxides and hydroperoxides (*Z*,*E* isomers and *E*,*E* isomers); (A–C) peaks from keto groups or alkyl-furanones [64]. For details about the region 3.3–5.3 ppm, see Supplementary Materials (Figure S2).

A representative NMR profile of the main lipid species present in the extract of a raw pasta sample is reported in Figure 2. The NMR spectra of different pasta samples showed very similar profiles, regardless of their composition, the most important observable differences being associated with signal intensities rather than different chemical species. It is worth noting that no observable signal from carbonyl groups of free fatty acids nor aldehydes could be found at low fields in the NMR spectra, suggesting that the majority of signals deriving from variably oxidized lipid species can be mostly ascribed to primary oxidation products (i.e., conjugated dienes systems as hydroperoxides, alcohols, keto-acids, and epoxides). An overlap of NMR peaks due to these species is clearly visible in the spectral regions 3.3–5.3 ppm (Figure 2 and Figure S2). Other relevant signals can be observed from 5.5–6.6 ppm and at about 7.5–8.0 ppm (Figure 2). According to the literature, the most representative signals from primary lipid oxidation products are expected to appear in the 3.3–5.3 ppm spectral region (Figure S2) [62,63,64,65].

**Figure 3:**
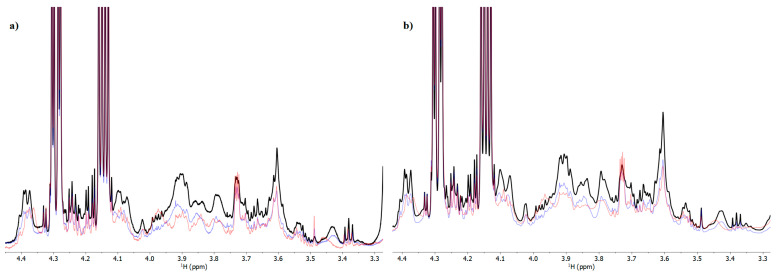
(**a**) ^1^H NMR spectra of CP (black) compared with RGP (blue) and SP (red), at the beginning of the conservation period. (**b**) Comparison between CP (black), RGP (blue), and SP (red) after 75 days of storage.

No distinct trends within the same treatment, as a function of storage duration, could be found by comparing the NMR spectra of the lipid extracts of the CP, RGP, and SP samples. Since samples were examined after up to 75 days of storage, an increase in at least some oxidized species over time would be expected. However, experimental evidence (Figure 3) demonstrates that primary oxidation of lipids is present in pasta at the same level across time, with very tiny sample-dependent changes, ruling out any accumulating phenomena of oxidized lipids (Figure S3). When the differently formulated pasta samples were compared at the same storage time, CP always had higher amounts of oxidized lipid moieties than SP and RGP, the latter two showing almost identical concentrations of such species.

**Figure 4:**
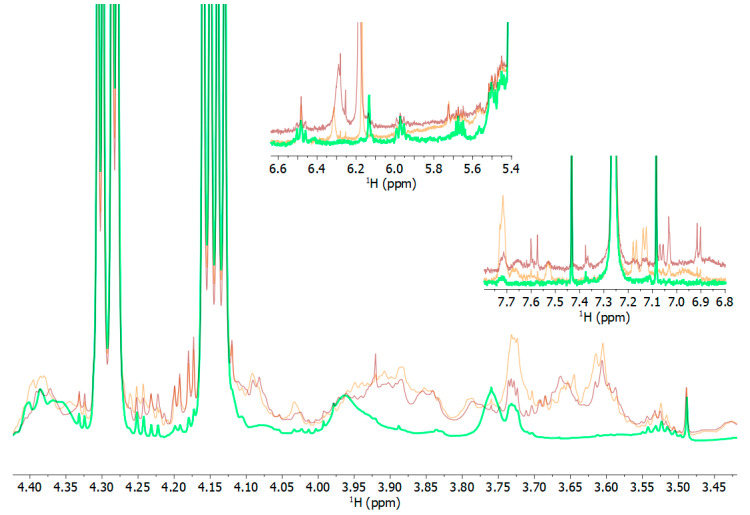
Comparison between selected spectral regions of ^1^H NMR spectra, where oxidized lipid species are expected to appear, in the raw ingredients semolina (orange), wholemeal semolina (brown), and wheat germ (green).

The comparison of the NMR spectra of pasta raw materials, namely semolina, wholemeal semolina, and wheat germ (Figure 4), revealed that the spectral region in which primary lipid oxidation products occur had significantly higher intensities in semolina and wholemeal semolina and lower intensities in raw germ. At the beginning of the storage time, this region of NMR spectra of the different pasta samples (Figure 3a) revealed that CP (pasta made with semolina) had a higher intensity than RGP (pasta made with semolina, wholemeal semolina, and raw germ) and SP (made with semolina, fermented wheat germ, and wholemeal semolina). Despite an increase in lipids from wheat germ, oxidation products in semolina and wholemeal semolina were lower after these ingredients were combined to make pasta with the wheat germ. Furthermore, the residual lipase activity found in SP sample (Table 6) did not lead to an increase in oxidized lipids at the end of the storage time. This suggests that wheat germ may have a free radical scavenging action against lipid oxidation, most likely due to higher concentration of antioxidant compounds in RGP and SP than in CP, as shown in Table 4. The intake of antioxidant-rich foods, such as fruits and vegetables, was found to play a protective role, preventing from oxidative stress-related and cardiovascular disease [66,67]. The fermentation process carried out on wheat germ and wholemeal semolina in sourdough did not markedly affect the oxidation status of fresh pasta at the molecular level, despite the observed increase in antioxidant activity compared to the RGP sample (Table 4).

The results of total lipids quantification and GC analysis of FAME, which were performed on all pasta samples at varied storage periods, should be examined to further support the conclusions stated above based on NMR observations. Although we measured a somewhat lower percentage of oleic acid, the fatty acid content of CP substantially approaches that of past scientific investigations on semolina flour [68]. Regardless of the duration of storage, all samples maintained a consistent level of total lipids, suggesting minimal or no degradation resulting in lipid loss, although RGP and SP initially contained more lipids than CP (Table 1). In particular, no significant deviation from the original FAME profile was observed for each pasta formulation as a function of storage time (Figure S4). It is important to notice the long-term stability of polyunsaturated fatty acids over time, especially those more vulnerable to oxidation, such as linoleic (LA) and α-linolenic acid (ALA). Concerning the importance of the dietary intake of polyunsaturated fatty acids, some studies have associated an adequate intake of LA with health benefits, such as the reduced risk of cardiovascular diseases [6]. It should be acknowledged in this context that some minor variations were present between replicates and that this within-group variability is a fundamental property of the system that was not consistently or progressively impacted by lipid oxidation processes during storage. All of the information regarding FAME profiles of pasta samples and ingredients are reported in the supplementary materials (Tables S4–S7).

### 3.8. Sensory Analysis

Sensory analysis was carried out on fresh pasta to study the impact of fermented and unfermented germ and bran on consumer sensory acceptability. The test was conducted just after pasta production (time 0) and repeated after 30 and 60 days of storage, in order to evaluate the shelf life of pasta samples. Mean scores of sensory properties are reported in Table 7. The analysis of data indicates that RGP differed from the other samples (CP and SP) for all the sensory properties considered, and at all times, except for the overall liking at time 0, that was similar for the three samples. Observing the box plot of the overall liking, reported in Figure S5, a wider score distribution at time 0 for CP and SP samples and a tendency for higher scores, with respect to the RGP sample, was shown. Moreover, the scores of RGP samples, for all the sensory properties considered, always showed a tendency toward lower values, as indicated by the box plot in Figure S5 and according to the ANOVA results reported in Table 7. 

The literature results on sensory properties of foods prepared with the use of wheat germ and/or wheat bran are somewhat controversial. Unpleasant sensory properties were the main reason for not eating whole grain pasta, as observed by Laureati et al. [11], and a decrease in the overall acceptability was found in fresh pasta made with wheat bran and wheat germ [69], and in noodles made with wheat germ [37]. On the contrary, Sun et al. [70] improved the acceptability of steamed bread with the addition of wheat germ, and Pinarli et al. [36] did not find any significant differences in the texture and overall score between pasta with added wheat germ and control pasta. In this study, pasta with raw GWS was less appreciated than pasta with fermented GWS, and this proved that a processing modification can improve the liking of pasta containing wheat germ and bran. Moreover, the acceptability score of pasta with fermented germ was similar to the score of pasta with semolina, which represents the pasta usually consumed. Finally, consumers rated CP and SP samples positively even after 60 days of storage, whereas the score of RGP sample decreased below 6 after 60 days. The results of sensory acceptability confirmed the absence of rancid and bitter compounds in pasta containing fermented and unfermented wheat germ.

## 4. Conclusions

This study highlights the properties of fresh pasta fortified with un-fermented (RGP) and fermented (SP) wheat germ and wholemeal semolina. Overall, the inclusion of wheat germ and wholemeal semolina, both fermented and not, had positive effects, particularly on the nutritional properties: proteins, lipids, vitamin E, dietary fibers, protein digestibility, and antioxidant compounds increased in both samples in comparison with the control sample (CP). As far as the negative effects were concerned, an increase in phytic acid, an anti-nutritional factor, was observed in RGP but not in SP, where the amount of phytic acid was similar to CP, as an outcome of the fermentation process. Moreover, the fermentation worked positively on some other properties of SP: the total phenolic content and the antioxidant activity were higher in SP than in RGP; the acceptability scores of the sensory properties (overall liking, smell, taste, etc.) were higher in SP than in RGP, and were similar to CP. In terms of pasta shelf life, the presence of lipids from wheat germ is expected to be a valid condition for triggering the rancidity process and decreasing pasta quality. However, all the analyses carried out in this study highlighted the high quality of pasta containing wheat germ, both fermented and not, even after several months of storage at low temperature and modified atmosphere. The study of lipid oxidation phenomena, by means of the ^1^H NMR technique, demonstrated that primary oxidation of lipids was higher in CP than in RGP and SP, and thus a protective effect of wheat germ has been postulated. 

## Figures and Tables

**Table 1:** Physicochemical properties of raw pasta samples.

	Moisture	Proteins	Ashes	Lipids	pH	TTA	Color			ΔE
Samples	(g/100 g)	(g/100 g d.b.)				(mL NaOH)	L*	b*	a*	
CP	24.09 ± 2.49 ^c^	11.82 ± 0.29 ^b^	0.84 ± 0.06 ^c^	1.17 ± 0.08 ^b^	6.84 ± 0.04 ^a^	1.43 ± 0.10 ^c^	66.94 ± 1.62 ^a^	27.24 ± 0.22 ^a^	1.06 ± 1.23 ^c^	
RGP	25.47 ± 1.38 ^b^	13.07 ± 0.37 ^a^	1.27 ± 0.06 ^b^	2.06 ± 0.16 ^a^	6.83 ± 0.06 ^a^	1.98 ± 0.19 ^b^	62.68 ± 1.48 ^c^	26.01 ± 0.38 ^b^	3.45 ± 1.51 ^b^	5.04
SP	26.32 ± 2.20 ^a^	13.31 ± 0.23 ^a^	1.31 ± 0.08 ^a^	2.09 ± 0.06 ^a^	4.82 ± 0.07 ^b^	7.72 ± 0.81 ^a^	64.56 ± 1.96 ^b^	25.01 ± 0.41 ^c^	3.63 ± 1.48 ^a^	4.15

Mean of, at least, three replicates ± standard deviation. d.b. = dry basis. Values in the same column with different superscript letters differ significantly (*p* ≤ 0.05).

**Table 2:** Cooking quality of pasta samples.

	OCT ^1^	CL ^2^	SI ^3^	WAI ^4^
	(min.)	(%)	(%)	(%)
CP	4.8 ± 0.32 ^a^	16.11 ± 0.74 ^c^	2.73 ± 0.01 ^a^	90.22 ± 0.61 ^a^
RGP	3.9 ± 0.60 ^ab^	17.65 ± 0.86 ^b^	2.61 ± 0.03 ^b^	82.53 ± 4.78 ^b^
SP	3.3 ± 0.52 ^bc^	18.72 ± 0.94 ^a^	2.51 ± 0.05 ^c^	71.38 ± 7.84 ^c^

^1^ Optimal Cooking Time (minutes). ^2^ Cooking Loss (grams of solids per 100 g of pasta as is). ^3^ Swelling Index (grams of water after cooking per gram of dry pasta). ^4^ Water absorption Index (grams of absorbed water per 100 g of raw pasta). Mean of, at least, four replicates ± standard deviation. Values in the same column with different superscript letters differ significantly (*p* ≤ 0.05).

**Table 3:** Texture profile analysis of cooked pasta samples.

	Hardness	Adhesiveness	Springiness	Cohesiveness	Gumminess	Chewiness
	N	N × s	s	n/a	N	N
CP	11.82 ± 1.14 ^a^	−0.03 ± 0.01 ^a^	0.91 ± 0.02 ^a^	0.62 ± 0.02 ^a^	7.21 ± 0.65 ^a^	6.56 ± 0.55 ^a^
RGP	11.81 ± 1.59 ^a^	−0.03 ± 0.01 ^a^	0.87 ± 0.05 ^b^	0.63 ± 0.01 ^a^	7.49 ± 0.62 ^a^	6.51 ± 0.19 ^a^
SP	10.86 ± 0.60 ^b^	−0.09 ± 0.04 ^b^	0.82 ± 0.07 ^c^	0.57 ± 0.02 ^b^	6.40 ± 0.15 ^b^	5.20 ± 0.51 ^b^

Gumminess is calculated as Hardness × Cohesiveness. Chewiness is calculated as Gumminess × Springiness. Mean of, at least, four replicates ± standard deviation. Values in the same column with different superscript letters differ significantly (*p* ≤ 0.05).

**Table 4:** Nutritional properties of cooked pasta samples.

	CP	RGP	SP
Protein Digestibility (%) *	49.58 ^c^	57.67 ^a^	54.10 ^b^
Protein Availability (g/100 pasta d.b.)	5.88 ^c^	7.59 ^a^	7.22 ^b^
Phytic acid (g/100 g d.b.)	0.17 ± 0.01 ^c^	0.36 ± 0.01 ^a^	0.18 ± 0.01 ^b^
Total dietary fiber (g/100 g d.b.)	6.32 ± 0.12 ^c^	8.34 ± 0.14 ^b^	8.66 ± 0.04 ^a^
Total Phenolic Content (mg/100 g d.b.)	46.79 ± 1.13 ^c^	60.47 ± 7.27 ^b^	76.77 ± 5.76 ^a^
Antioxidant activity (%) **	2.86 ± 0.83 ^c^	4.42 ± 0.54 ^b^	7.37 ± 0.72 ^a^

* Amount of proteins digested over total protein content. ** Percentage of discoloration of DPPH solution of pasta sample respect to a blank sample. d.b. = dry basis. Mean of, at least, three replicates ± standard deviation. Values in the same row with different superscript letters differ significantly (*p* ≤ 0.05).

**Table 5:** Content of tocols in raw materials and pasta samples (raw and cooked).

		α-TP	α–TT	β-TP	β-TT	Total
		(μg/g d.b.)	(μg/g d.b.)	(μg/g d.b.)	(μg/g d.b.)	(μg/g d.b.)
Raw materials	Semolina	10.85 ± 3.42	2.89 ± 0.80	10.14 ± 3.23	12.8 ± 0.98	36.68 ± 6.15
	Wholemeal semolina	26.71 ± 2.15	2.00 ± 1.02	17.62 ± 1.62	18.10 ± 2.47	64.43 ± 9.04
	GWS	30.24 ± 3.46	6.00 ± 2.95	25.68 ± 1.01	15.95 ± 2.95	77.87 ± 8.26
	Sourdough	28.52 ± 5.70	2.63 ± 0.53	29.97 ± 5.09	16.38 ± 3.03	77.50 ± 12.35
Raw pasta	CP	7.96 ± 1.32 ^b^	0.36 ± 0.05 ^b^	6.88 ± 1.11 ^c^	6.93 ± 1.03 ^b^	22.13 ± 2.63 ^b^
	RGP	27.69 ± 4.44 ^a^	0.73 ± 0.15 ^a^	17.32 ± 0.34 ^b^	8.79 ± 0.61 ^a^	54.53 ± 4.31 ^a^
	SP	23.34 ± 2.58 ^a^	0.73 ± 0.18 ^a^	23.83 ± 3.36 ^a^	7.97 ± 0.84 ^a^	55.86 ± 5.03 ^a^
Cooked pasta	CP	4.02 ± 1.72 ^a^	0.38 ± 0.09 ^a^	5.67 ± 0.79 ^b^	6.16 ± 1.24 ^a^	16.23 ± 2.52 ^c^
	RGP	4.36 ± 1.78 ^a^	0.40 ± 0.12 ^a^	8.42 ± 1.81 ^b^	5.90 ± 1.63 ^a^	19.09 ± 5.10 ^b^
	SP	6.31 ± 1.36 ^a^	0.20 ± 0.07 ^b^	22.58 ± 3.26 ^a^	7.08 ± 0.96 ^a^	36.17 ± 5.53 ^a^

α-TP = α-tocopherol; α-TT = α-tocotrienol; β-TP = β-tocopherol; β-TT = β-tocotrienol. d.b. = dry basis. GWS = blend of wheat germ and wholemeal semolina; CP = Control Pasta; RGP = Raw Germ Pasta; SP = Sourdough Pasta. Mean of three replicates ± standard deviation. For raw and cooked pasta, values in the same column with different superscript letters differ significantly (*p* ≤ 0.05).

**Table 6:** Residual lipase and lipoxygenase activity.

	Lipase (%)	Lipoxygenase (%)
GWS	100 ^a^	100 ^b^
S	31.00 ± 6.08	59.11 ± 10.93
CP	0	20.04 ± 3.57
RGP	0	27.13 ± 4.14
SP	18.00 ± 3.65	29.88 ± 5.57

GWS: blend of germ and wholemeal semolina. S: Sourdough. CP: Control pasta. RGP: Raw germ pasta. SP: Sourdough pasta. ^a^ 21.76 ± 1.65 μmol NaOH×g (d.b.) × 60 min. ^b^ 20.77 ± 3.71 μmol conjugated dienes × g (d.b.) × min.

**Table 7:** Mean acceptability scores for sensory characteristics of pasta samples at 0, 30, and 60 days of storage.

Samples	Days of Storage	Appearance	Smell	Taste	Texture	Overall Liking
CP	0	7.70 ± 1.18 ^aA^	7.11 ± 1.03 ^aA^	7.13 ± 1.05 ^aA^	7.00 ± 1.09 ^aA^	6.88 ± 1.03 ^aA^
RGP	0	6.76 ± 1.45 ^bA^	6.25 ± 0.93 ^bA^	6.09 ± 1.78 ^bA^	6.43 ± 1.31 ^bA^	6.60 ± 1.28 ^aA^
SP	0	7.70 ± 1.18 ^aA^	7.09 ± 1.02 ^aA^	7.03 ± 1.14 ^aA^	7.01 ± 1.10 ^aA^	7.00 ± 1.09 ^aA^
CP	30	7.11 ± 0.88 ^aA^	6.62 ± 0.72 ^aB^	6.62 ± 0.69 ^aA^	6.58 ± 0.63 ^aA^	6.66 ± 0.75 ^aA^
RGP	30	6.17 ± 1.43 ^bAB^	5.96 ± 0.89 ^bAB^	5.62 ± 1.62 ^bAB^	6.01 ± 1.15 ^bAB^	5.60 ± 1.29 ^bB^
SP	30	6.98 ± 0.94 ^aB^	6.54 ± 0.70 ^aB^	6.49 ± 0.88 ^aA^	6.45 ± 0.67 ^bB^	6.56 ± 0.76 ^aA^
CP	60	7.64 ± 1.03 ^aA^	6.96 ± 0.87 ^aAB^	6.96 ± 0.89 ^aA^	6.94 ± 0.94 ^aA^	6.72 ± 0.98 ^aA^
RGP	60	5.82 ± 1.24 ^bB^	5.58 ± 1.05 ^bB^	5.27 ± 1.47 ^bB^	5.72 ± 0.96 ^bB^	5.58 ± 1.11 ^bB^
SP	60	7.31 ± 1.14 ^aAB^	6.88 ± 0.86 ^aAB^	6.76 ± 1.06 ^aA^	6.76 ± 0.99 ^aAB^	6.70 ± 0.83 ^aA^

In the same column, for the same days, different lowercase letters indicate a significant difference (*p* ≤ 0.001) between samples. In the same column, for the same sample, different uppercase letters indicate a significant difference (*p* ≤ 0.001) along storage.

## Data Availability

The datasets generated for this study are available on request to the corresponding author.

## Supplementary Materials

The following supporting information can be downloaded at: https://www.mdpi.com/article/10.3390/foods12142641/s1, Table S1: Physicochemical composition of raw materials; Table S2: Viable counts of LAB, yeast, mould and aerobic bacteria; Table S3: Yeasts and LAB species found in sourdough; Table S4: Fatty acid profile of raw materials; Tables S5–S7: Complete fatty acid profile of Control Pasta (CP), Raw Germ Pasta (RGP), Sourdough Pasta (SP). Figure S1: Plate assay for the lipolytic activity of the yeast strains. Figure S2: ^1^H NMR spectrum of the lipid extract of a raw pasta sample showing the most representative signals of primary oxidation lipid products; Figure S3: The spectra ^1^H NMR of an RGP sample across storage time. Figure S4: Fatty acid profile of raw pasta samples during storage; Figure S5: Box Plot of the overall liking for the consumer acceptance test using a nine-point hedonic scale.

## References

[1] Boukid F., Folloni S., Ranieri R., Vittadini E. (2018). A compendium of wheat germ: Separation, stabilization and food applications. Trends Food Sci. Tech..

[2] Brandolini A., Hidalgo A. (2012). Wheat germ: Not only a by-product. Int. J. Food Sci. Nutr..

[3] Hemdane S., Jacobs P.J., Dornez E., Verspreet J., Delcour J.A., Courtin C.M. (2016). Wheat (*Triticum aestivum* L.) bran in bread making: A critical review. Compr. Rev. Food Sci. Food Saf..

[4] Stevenson L.E.O., Phillips F., O’sullivan K., Walton J. (2012). Wheat bran: Its composition and benefits to health, a European perspective. Int. J. Food Sci. Nutr..

[5] Hu Y., Willett W.C., Manson J.A.E., Rosner B., Hu F.B., Sun Q. (2022). Intake of whole grain foods and risk of coronary heart disease in US men and women. BMC Med..

[6] Marangoni F., Agostoni C., Borghi C., Catapano A.L., Cena H., Ghiselli A., La Vecchia C., Lercker G., Manzato E., Pirillo A. (2020). Dietary linoleic acid and human health: Focus on cardiovascular and cardiometabolic effects. Atherosclerosis.

[7] Ghafoor K., Özcan M.M., AL-Juhaımı F., Babıker E.E., Sarker Z.I., Ahmed I.A.M., Ahmed M.A. (2017). Nutritional composition, extraction, and utilization of wheat germ oil: A review. Eur. J. Lipid Sci. Tech..

[8] de Punder K., Pruimboom L. (2013). The dietary intake of wheat and other cereal grains and their role in inflammation. Nutrients.

[9] Bin Q., Jiang D., Cho I.H., Peterson D.G. (2012). Chemical markers for bitterness in wheat bread. Flavour Fagr. J..

[10] Heiniö R.L., Noort M.W.J., Katina K., Alam S.A., Sozer N., De Kock H.L., Hersleth M., Poutanen K. (2016). Sensory characteristics of wholegrain and bran-rich cereal foods—A review. Trends Food Sci. Tech..

[11] Laureati M., Conte A., Padalino L., Del Nobile M.A., Pagliarini E. (2016). Effect of fiber information on consumer’s expectation and liking of wheat bran enriched pasta. J. Sens. Stud..

[12] Poutanen K., Flander L., Katina K. (2009). Sourdough and cereal fermentation in a nutritional perspective. Food Microbial..

[13] Pontonio E., Lorusso A., Gobbetti M., Rizzello C.G. (2017). Use of fermented milling by-products as functional ingredient to develop a low-glycaemic index bread. J. Cereal Sci..

[14] Bayat E., Moosavi-Nasab M., Fazaeli M., Majdinasab M., Mirzapour-Kouhdasht A., Garcia-Vaquero M. (2022). Wheat germ fermentation with *Saccharomyces cerevisiae* and *Lactobacillus plantarum*: Process optimization for enhanced composition and antioxidant properties in vitro. Foods.

[15] Marti A., Torri L., Casiraghi M.C., Franzetti L., Limbo S., Morandin F., Quaglia L., Pagani M.A. (2014). Wheat germ stabilization by heat-treatment or sourdough fermentation: Effects on dough rheology and bread properties. LWT-Food Sci. Technol..

[16] Tovar L.E.R., Gänzle M.G. (2021). Degradation of wheat germ agglutinin during sourdough fermentation. Foods.

[17] Rizzello C.G., Nionelli L., Coda R., Di Cagno R., Gobbetti M. (2010). Use of sourdough fermented wheat germ for enhancing the nutritional, texture and sensory characteristics of the white bread. Eur. Food Res. Technol..

[18] AACC (2010). Approved Methods of Analysis.

[19] Fois S., Campus M., Piu P.P., Siliani S., Sanna M., Roggio T., Catzeddu P. (2019). Fresh pasta manufactured with fermented whole wheat semolina: Physicochemical, sensorial, and nutritional properties. Foods.

[20] Folch J., Lees M., Sloane Stanley G.H. (1957). A simple method for the isolation and purification of total lipids from animal tissues. J. Biol. Chem..

[21] Teterycz D., Sobota A., Starek A. (2022). Possibility of using wheat germ and wheat germ protein isolate for high-protein pasta production. Cereal Chem..

[22] Fois S., Piu P.P., Sanna M., Roggio T., Catzeddu P. (2018). Starch digestibility and properties of fresh pasta made with semolina-based liquid sourdough. LWT-Food Sci. Technol..

[23] Padalino L., Mastromatteo M., Lecce L., Spinelli S., Contò F., Del Nobile M.A. (2014). Effect of durum wheat cultivars on physico-chemical and sensory properties of spaghetti. J. Sci. Food Agric..

[24] Manthey F.A., Dick T. (2012). Assessment of probe type for measuring pasta texture. Cereal Foods World.

[25] Pasini G., Simonato B., Giannattasio M., Peruffo A.D.B., Curioni A. (2001). Modifications of wheat flour proteins during in vitro digestion of bread dough, crumb, and crust: An electrophoretic and immunological study. J. Agric. Food Chem..

[26] De Marco E.R., Steffolani M.E., Martínez C.S., León A.E. (2014). Effects of spirulina biomass on the technological and nutritional quality of bread wheat pasta. LWT-Food Sci. Technol..

[27] Singleton V.L., Rossi J.A. (1965). Colorimetry of total phenolics with phosphomolybdic-phosphotungstic acid reagents. Am. J. Enol. Viticult..

[28] Boroski M., de Aguiar A.C., Boeing J.S., Rotta E.M., Wibby C.L., Bonafé E.G., de Souza N.E., Visentainer J.V. (2011). Enhancement of pasta antioxidant activity with oregano and carrot leaf. Food Chem..

[29] Tolouie H., Mohammadifar M.A., Ghomi H., Yaghoubi A.S., Hashemi M. (2018). The impact of atmospheric cold plasma treatment on inactivation of lipase and lipoxygenase of wheat germs. Innov. Food Sci. Emerg. Tech..

[30] Hidalgo A., Brandolini A. (2012). Lipoxygenase activity in wholemeal flours from *Triticum monococcum*, *Triticum turgidum* and *Triticum aestivum*. Food Chem..

[31] Siji J. (2011). Analysis of fat-soluble vitamins from food matrix for nutrition labeling. Agilent Application Note.

[32] Nurit E., Lyan B., Pujos-Guillot E., Branlard G., ve Piquet A. (2016). Change in B and E vitamin and lutein, β-sitosterol contents in industrial milling fractions and during toasted bread production. J. Cereal Sci..

[33] Karakas F.P., Keskin C.N., Agil F., Zencirci N. (2021). Profiles of vitamin B and E in wheat grass and grain of einkorn (*Triticum monococcum* spp. *monococcum*), emmer (*Triticum dicoccum* ssp. *dicoccum Schrank*.), durum (*Triticum durum* Desf.), and bread wheat (*Triticum aestivum* L.) cultivars by LC-ESI-MS/MS analysis. J. Cereal Sci..

[34] Siliani S., Melis R., Loi B., Guala I., Baroli M., Sanna R., Uzzau S., Roggio T., Addis M.F., Anedda R. (2016). Influence of seasonal and environmental patterns on the lipid content and fatty acid profiles in gonads of the edible sea urchin *Paracentrotus lividus* from Sardinia. Mar. Environ. Res..

[35] Melis R., Vitangeli I., Anedda R. (2022). Effect of fish diet and cooking mode on the composition and microstructure of ready-to-eat fish fillets of gilthead sea bream (*Sparus aurata*) juveniles. J. Food Compos. Anal..

[36] Pınarlı I., Öner M.D., İbanoğlu Ş. (2004). Effect of wheat germ addition on the microbiological quality, in vitro protein digestibility, and gelatinization behavior of macaroni. Eur. Food Res. Technol..

[37] Aktaş K., Bilgiçli N., Levent H. (2015). Influence of wheat germ and β-glucan on some chemical and sensory properties of Turkish noodle. J. Food Sci. Technol..

[38] Drabińska N., Nogueira M., Ciska E., Jelen H. (2022). Effect of drying and broccoli leaves incorporation on the nutritional quality of durum wheat pasta. Pol. J. Food Nutr. Sci..

[39] Fuad T., Prabhasankar P. (2010). Role of ingredients in pasta product quality: A review on recent developments. Crit. Rev. Food Sci. Nutr..

[40] Tarzi B.G., Shakeri V., Ghavami M. (2012). Quality evaluation of pasta enriched with heated and unheated wheat germ during storage. Adv. Environ. Biol..

[41] Matsuo R.R., Dexter J.E., Boudreau A., Daun J.K. (1986). The role of lipids in determining spaghetti cooking quality. Cereal Chem..

[42] Aravind N., Sissons M., Egan N., Fellows C. (2012). Effect of insoluble dietary fibre addition on technological, sensory, and structural properties of durum wheat spaghetti. Food Chem..

[43] Vignola M.B., Bustos M.C., Pérez G.T. (2018). In vitro dialyzability of essential minerals from white and whole grain pasta. Food Chem..

[44] Östman E.M., Nilsson M., Elmståhl H.L., Molin G., Björck I.M.E. (2002). On the effect of lactic acid on blood glucose and insulin responses to cereal products: Mechanistic studies in healthy subjects and in vitro. J. Cereal Sci..

[45] Schettino R., Pontonio E., Rizzello C.G. (2019). Use of fermented hemp, chickpea and milling by-products to improve the nutritional value of semolina pasta. Foods.

[46] Labuckas D.O., Maestri D.M., Perelló M., Martínez M.L., Lamarque A.L. (2008). Phenolics from walnut (*Juglans regia* L.) kernels: Antioxidant activity and interactions with proteins. Food Chem..

[47] Sze-Tao K.W.C., Sathe S.K. (2000). Walnuts (*Juglans regia* L): Proximate composition, protein solubility, protein amino acid composition and protein in vitro digestibility. J. Sci. Food Agric..

[48] Gobbetti M., Rizzello C.G., Di Cagno R., De Angelis M. (2014). How the sourdough may affect the functional features of leavened baked goods. Food Microbiol..

[49] Hur S.J., Lee S.Y., Kim Y.C., Choi I., Kim G.B. (2014). Effect of fermentation on the antioxidant activity in plant-based foods. Food Chem..

[50] Barnes P.J. Cereal tocopherols. Proceedings of the 7th World Cereal and Bread Congress.

[51] Piironen V., Syvaoja E.L., Varo P., Salminen K., Koivistoinen P. (1986). Tocopherols and tocotrienols in Finnish foods: Vegetables, fruits, and berries. J. Agric. Food Chem..

[52] Nielsen M.M.L., Hansen Å. (2008). Stability of vitamin E in wheat flour and whole wheat flour during storage. Cereal Chem..

[53] Fardet A. (2010). New hypotheses for the health-protective mechanisms of whole-grain cereals: What is beyond fibre?. Nutr. Res. Rev..

[54] Fratianni A., Di Criscio T., Mignogna R., Panfili G. (2012). Carotenoids, tocols and retinols evolution during egg pasta–making processes. Food Chem..

[55] Ateş Sönmezoğlu Ö., Balkan A.S. (2014). Molecular and biochemical analysis of durum wheat genotypes to examine carotenoid pigment content and lipoxygenase enzyme activity. Cereal Res. Commun..

[56] Rose D.J., Pike O.A. (2006). A simple method to measure lipase activity in wheat and wheat bran as an estimation of storage quality. J. Am. Oil Chem. Soc..

[57] Bahal G., Sudha M.L., Ramasarma P.R. (2013). Wheat germ lipoxygenase: Its effect on dough rheology, microstructure, and bread making quality. Int. J. Food Prop..

[58] Arslan D., Demir K.M., Acar A., Arslan F.N. (2020). Investigation of wheat germ and oil characteristics with regard to different stabilization techniques. Food Technol. Biotech..

[59] Rizzello C.G., Nionelli L., Coda R., De Angelis M., Gobbetti M. (2010). Effect of sourdough fermentation on stabilisation, and chemical and nutritional characteristics of wheat germ. Food Chem..

[60] Syrokou M.K., Tziompra S., Psychogiou E.E., Mpisti S.D., Paramithiotis S., Bosnea L., Mataragas M., Skandamis P.N., Drosinos E.H. (2021). Technological and safety attributes of lactic acid bacteria and yeasts isolated from spontaneously fermented greek wheat sourdoughs. Microorganisms.

[61] Ciafardini G., Zullo B.A., Cioccia G., Iride A. (2006). Lipolytic activity of Williopsis californica and Saccharomyces cerevisiae in extra virgin olive oil. Int. J. Food Microbiol..

[62] Goicoechea E., Guillen M.D. (2010). Analysis of hydroperoxides, aldehydes and epoxides by ^1^H nuclear magnetic resonance in sunflower oil oxidized at 70 and 100 C. J. Agric. Food Chem..

[63] Martínez-Yusta A., Goicoechea E., Guillén M.D. (2014). A Review of thermo-oxidative degradation of food lipids studied by ^1^H NMR spectroscopy: Influence of degradative conditions and food lipid nature. Compr. Rev. Food Sci. Food Saf..

[64] Martin-Rubio A.S., Sopelana P., Ibargoitia M.L., Guillén M.D. (2021). 1H NMR study of the in vitro digestion of highly oxidized soybean oil and the effect of the presence of ovalbumin. Foods.

[65] Caño-Ochoa S.D., Ruiz-Aracama A., Guillén M.D. (2022). Alpha-Tocopherol, a powerful molecule, leads to the formation of oxylipins in polyunsaturated oils differently to the temperature increase: A detailed study by proton nuclear magnetic resonance of walnut oil oxidation. Antioxidants.

[66] Brighenti F., Valtuena S., Pellegrini N., Ardigo D., Del Rio D., Salvatore S., Piatti P.M., Serafini M., Zavaroni I. (2005). Total antioxidant capacity of the diet is inversely and independently related to plasma concentration of high-sensitivity C-reactive protein in adult Italian subjects. Brit. J. Nutr..

[67] Bazzano L.A., He J., Ogden L.G., Loria C.M., Vupputuri S., Myers L., Whelton P.K. (2002). Fruit and vegetable intake and risk of cardiovascular disease in US adults: The first National Health and Nutrition Examination Survey Epidemiologic Follow-up Study. Am. J. Clin. Nutr..

[68] Raczyk M., Polanowska K., Kruszewski B., Grygier A., Michałowska D. (2022). Effect of spirulina (*Arthrospira platensis*) supplementation on physical and chemical properties of semolina (*Triticum durum*) based fresh pasta. Molecules.

[69] Cankurtaran T., Bilgiçli N. (2019). Influence of wheat milling by-products on some physical and chemical properties of filled and unfilled fresh pasta. J. Food Sci. Technol..

[70] Sun R., Zhang Z., Hu X., Xing Q., Zhuo W. (2015). Effect of wheat germ flour addition on wheat flour, dough and Chinese steamed bread properties. J. Cereal Sci..

