# Supplementary material for: Quality Evaluation of Fresh Pasta Fortified with Sourdough Containing Wheat Germ and Wholemeal Semolina
Source: Foods. 2023 Jul 8;12(14):2641. doi: 10.3390/foods12142641 (PMC10378388; doi:10.3390/foods12142641)
Supplement: Supplementary file 1 [file foods-12-02641-s001.zip › foods-2486155-supplementary figures.pdf]

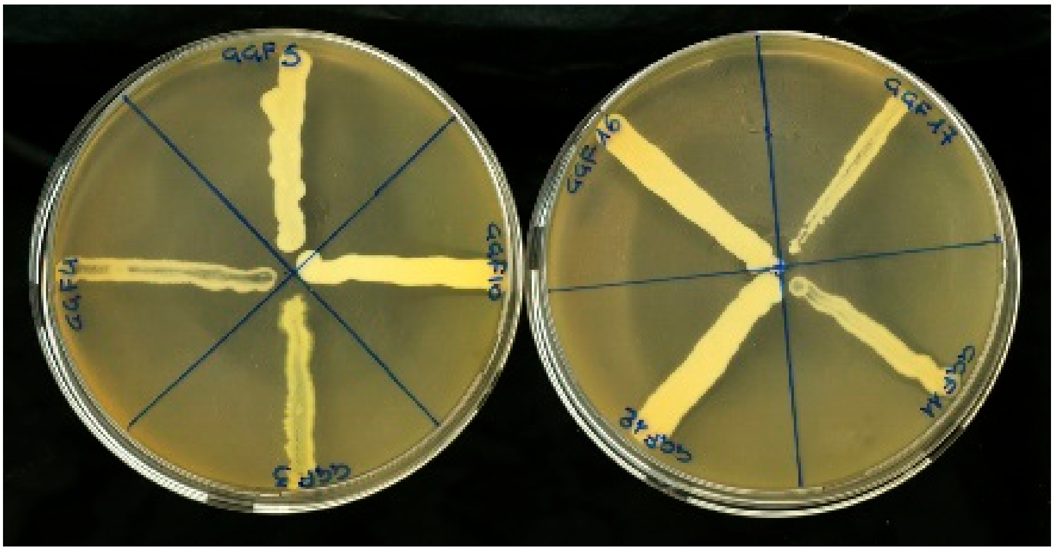

Figure S1. Plate assay for lipolytic activity of the yeast strains isolated from sourdough.

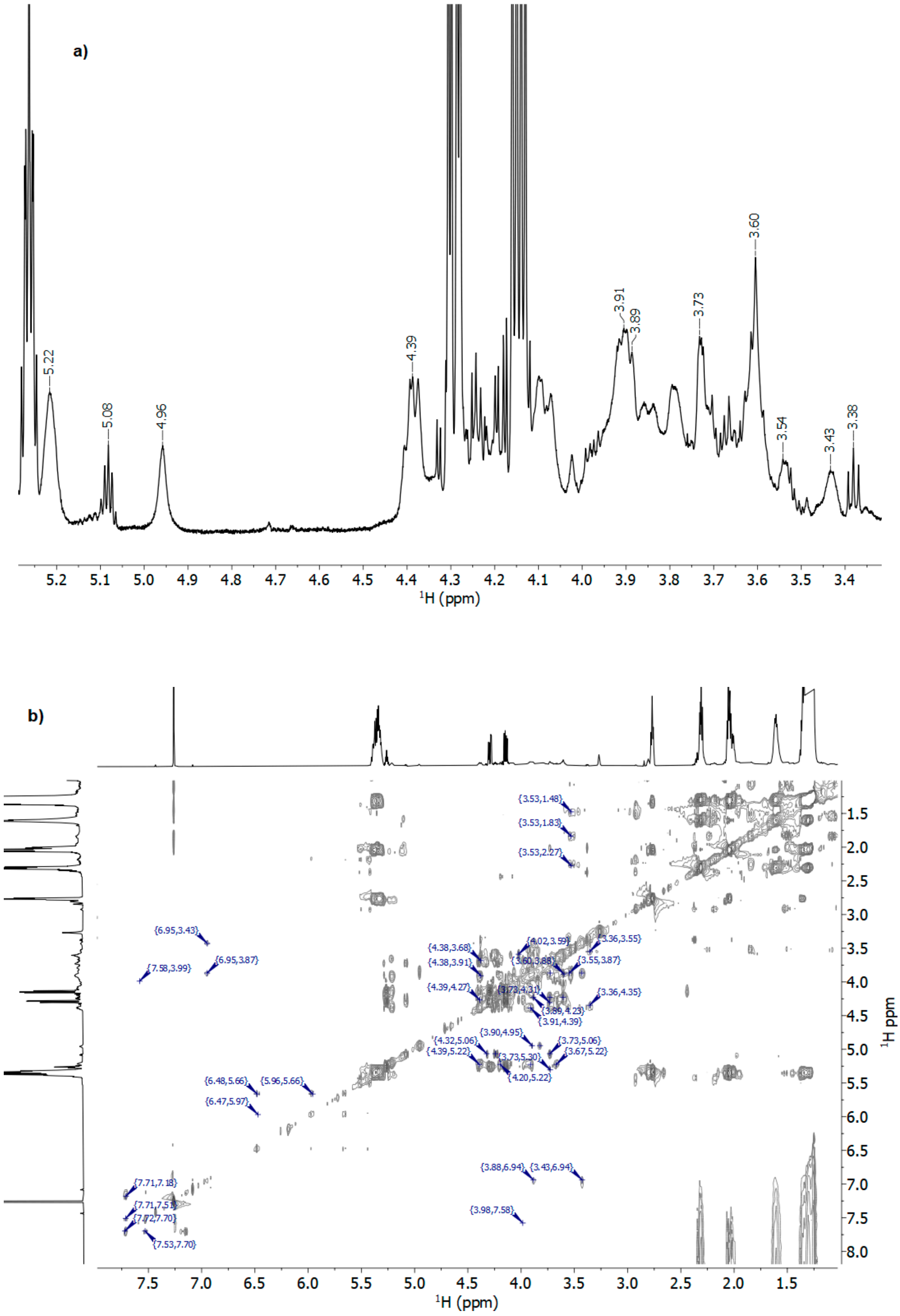
a)
5.22
5.08
4.96
4.39
3.91
3.89
3.73
3.60
3.54
3.43
3.38
5.2 5.1 5.0 4.9 4.8 4.7 4.6 4.5 4.4 4.3 4.2 4.1 4.0 3.9 3.8 3.7 3.6 3.5 3.4
^{1}H (ppm)
b)
{3.53,1.48}
{3.53,1.83}
{3.53,2.27}
{6.95,3.43}
{6.95,3.87}
{7.58,3.99}
{4.38,3.68}
{4.38,3.91}
{4.02,3.59}
{3.60,3.88}
{3.36,3.55}
{3.55,3.87}
{4.39,4.27}
{3.73,4.31}
{3.89,4.23}
{3.91,4.39}
{3.36,4.35}
{3.90,4.95}
{4.32,5.06}
{4.39,5.22}
{3.73,5.30}
{4.20,5.22}
{3.73,5.06}
{3.67,5.22}
{6.48,5.66}
{5.96,5.66}
{6.47,5.97}
{3.88,6.94}
{3.43,6.94}
{3.98,7.58}
{7.71,7.18}
{7.71,7.51}
{7.72,7.70}
{7.53,7.70}
1.5 2.0 2.5 3.0 3.5 4.0 4.5 5.0 5.5 6.0 6.5 7.0 7.5 8.0
^{1}H ppm
7.5 7.0 6.5 6.0 5.5 5.0 4.5 4.0 3.5 3.0 2.5 2.0 1.5
^{1}H (ppm)

Figure S2. a) expanded 3.3-5.3 ppm region of the typical ^{1}H NMR spectrum of the lipid extract (in $CDCl_3$) of a raw pasta sample. b) ^{1}H-^{1}H TOCSY NMR map of the same extract.

The chemical shifts (ppm) of the most representative signals of oxidized lipid species are reported above each peak and by peak peaking each relevant correlation on the 2D map. In this spectral range, the majority of signals are diagnostic signals for primary lipid oxidation compounds.

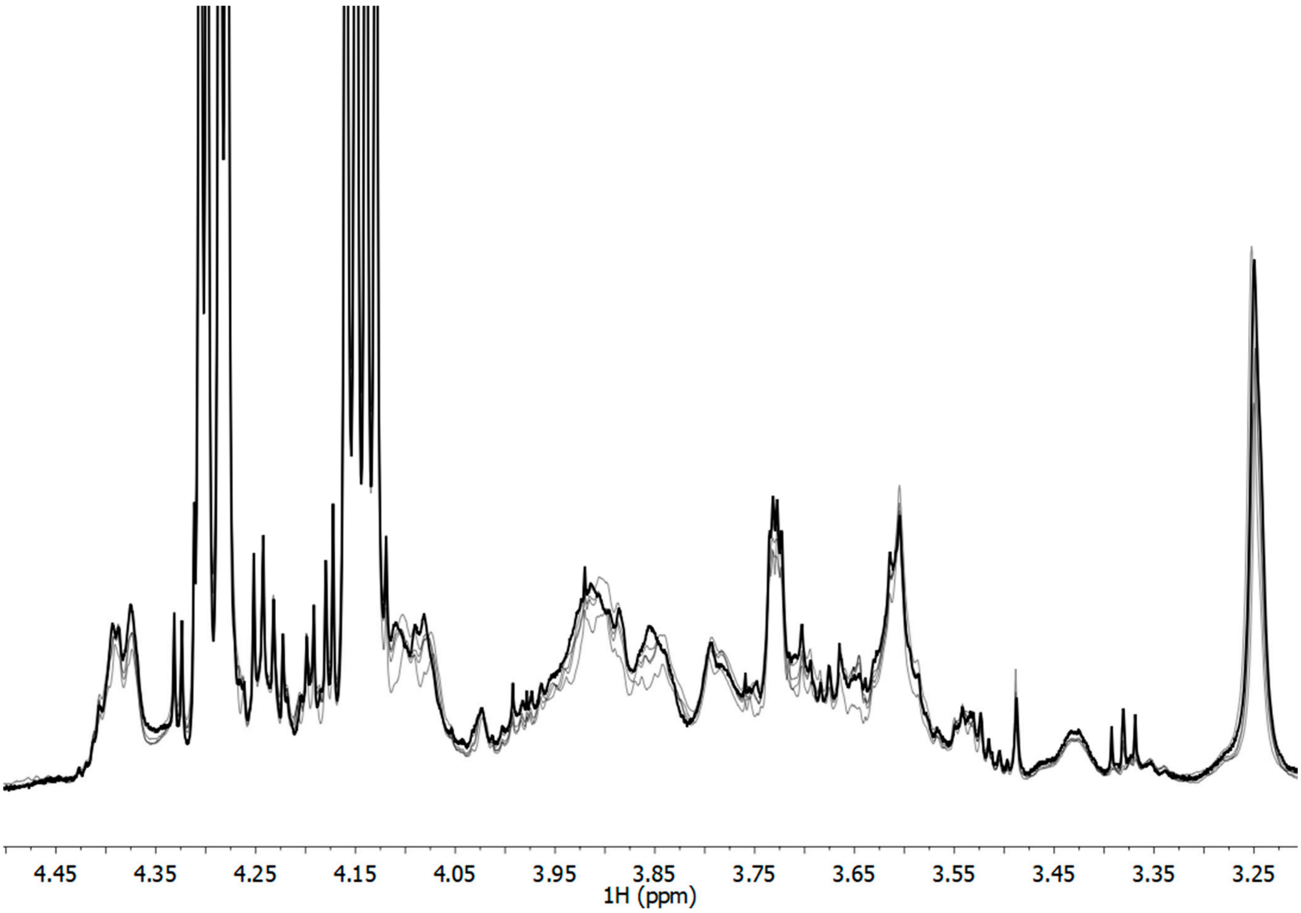

Figure S3. The spectra ^{1}H NMR of an RGP sample are displayed as an example of the conservation of lipid primary oxidation levels across storage time. Thickest line represents the raw RGP pasta sample at time zero, and other spectra the same pasta after 15, 35, 55 and 75 days of storage. The same trends were observed across storage time for all pasta compositions, therefore this stacked plot should be taken as a representative example of the behavior of oxidized species during storage.

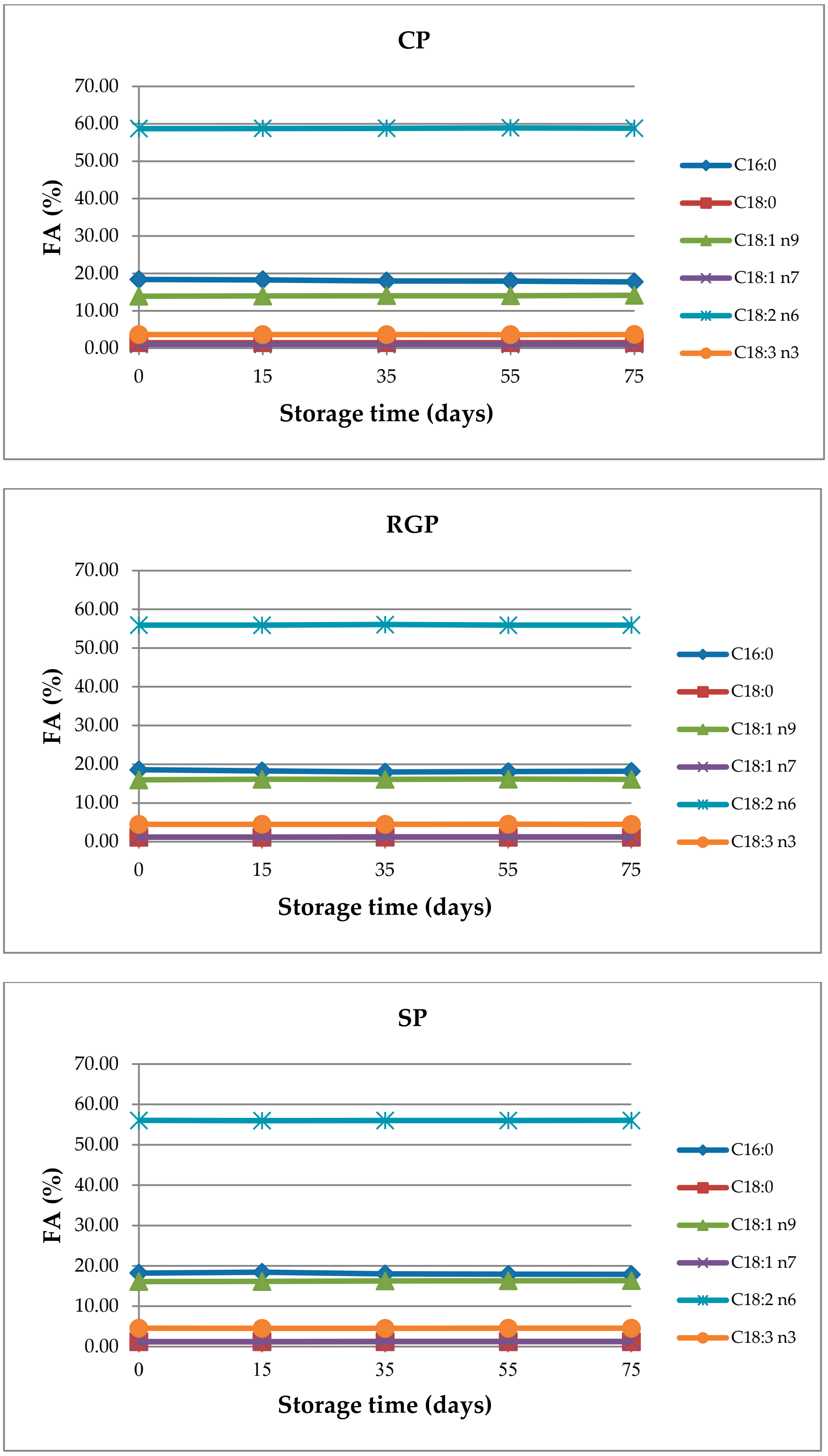

Figure S4. Fatty acid profile of raw pasta samples during storage at 5°C under modified atmosphere. Each fatty acid was expressed as percentage on the total fatty acids identified with GC analysis.

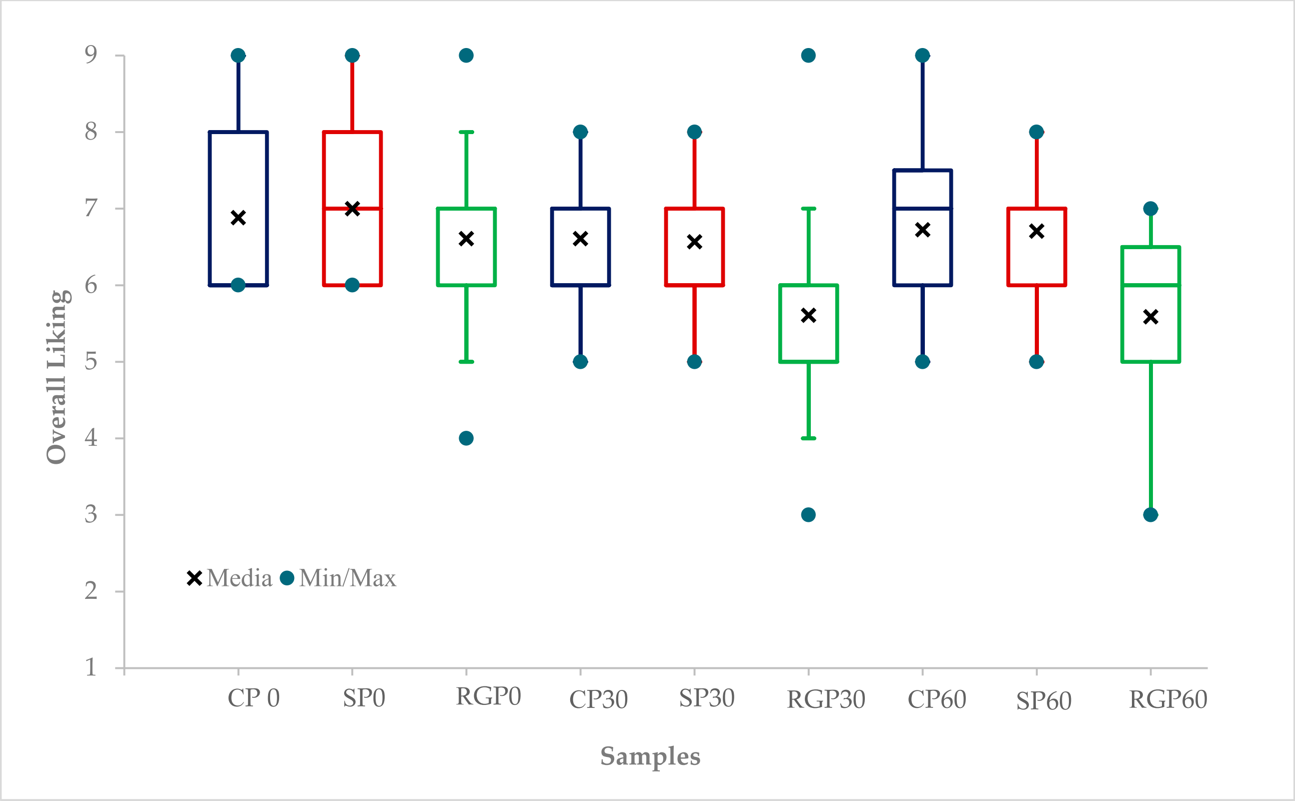

Figure S5. Box Plot of the overall liking for the consumer acceptance test using a nine-point hedonic scale.
