# Supplementary material for: Quality Evaluation of Fresh Pasta Fortified with Sourdough Containing Wheat Germ and Wholemeal Semolina
Source: Foods. 2023 Jul 8;12(14):2641. doi: 10.3390/foods12142641 (PMC10378388; doi:10.3390/foods12142641)
Supplement: Supplementary file 1 [file foods-12-02641-s001.zip › foods-2486155-supplementary tables.pdf]

Tabel S1. Physicochemical composition of raw materials used for pasta production

**Samples**	pH	**TTA** (mL NaOH 0.1N)	**Moisture** (g/100g)	**Ashes** (g/100g d.b.)	**Proteins** (g/100g d.b.)	**Gluten** (g/100g d.b.)
Wheat Germ	6,75(±0.02)	10,71(±0.11)	13,09(±0.03)	5,66(±0.02)	29,71(±0.76)	-
Wholemeal Semolina1	6,95(±0.03)	4,56(±0.02)	12,30(±0.02)	1,64(±0.04)	13,44(±0.24)	9,90(±0.18)
Wholemeal semolina2	6,54(±0.01)	4,31(±0.03)	14,87(±0.13)	1,51(±0.06)	13,25(±0.41)	9,58(±0.45)
Wholemeal Semolina3	6,54(±0.03)	4,88(±0.12)	12,50(±0.12)	1,54(±0.06)	13,26(±0.19)	9,20(±0.70)
Semolina1	6,71(±0.18)	2,41(±0.02)	13,73(±0.03)	0,90(±0.01)	11,47(±0.55)	7,92(±0.32)
Semolina2	6,52(±0.02)	2,28(±0.11)	13,77(±0.02)	0,83(±0.01)	11,32(±0.09)	8,52(±0.46)
Semolina3	6,55(±0.06)	2,44(±0.03)	13,31(±0.03)	0,77(±0.01)	11,16(±0.08)	8,02(±0.51)

Mean of, at least, three replicates ± standard deviation.

Table S2. Viable counts of LAB, yeast, mould and aerobic bacteria (cfu/g) in pasta samples stored at 5°C under modified atmosphere.

	0 gg			35 gg			75 gg			95 gg		
Samples	**LAB**	**Y&M**	**TAB**	**LAB**	**Y&M**	**TAB**	**LAB**	**Y&M**	**TAB**	**LAB**	**Y&M**	**TAB**
Raw wheat germ	$1.6x10^6$	$1.0x10^5$	$1.2x10^7$	n.d.	n.d.	n.d.	n.d.	n.d.	n.d.	n.d.	n.d.	n.d.
Sourdough	$1.0x10^9$	$1.7x10^6$	n.d.	n.d.	n.d.	n.d.	n.d.	n.d.	n.d.	n.d.	n.d.	n.d.
CP	$< 10^2$	$< 10^2$	$< 10^2$	$< 10^2$	$< 10^2$	$7 x 10^2$	$< 10^2$	$< 10^2$	$< 10^2$	$< 10^2$	$< 10^2$	$6 x 10^2$
RGP	$< 10^2$	$< 10^2$	$2.5 x 10^2$	$< 10^2$	$< 10^2$	60	$< 10^2$	$< 10^2$	60	$<10^2$	$<10^2$	$1 x 10^2$
SP	$< 10^2$	$< 10^2$	$1.5 x 10^2$	$< 10^2$	$< 10^2$	80	$< 10^2$	$< 10^2$	$1.2 x 10^2$	$<10^2$	$<10^2$	$1.5 x 10^2$

LAB = lactic acid bacteria; Y&M = yeast and mould; TAB = total aerobic bacteria; n.d. = not done

Table S3. Yeasts and LAB species found in sourdough

Kazachstania servazzii
Torulaspora delbrueckii
Pichia fermentas
Clavispora lusitaniae
Fructilactobacillus sanfranciscensis
Lactobacillus plantarum

Table S4. Fatty acid profile of raw materials used for pasta production

	**Palmitic acid** C16:0 (%)*	**Stearic acid** C18:0 (%)	**Oleic acid** C18:1 n9 (%)	***cis*-Vaccenic acid** C18:1 n7 (%)	**Linoleic acid** C18:2 n6 (%)	**α-Linolenic acid** C18:3 n3 (%)
Semolina	16.80 (±0.41)	1.38 (±0.02)	16.76 (±0.42)	1.09 (±0.04)	57.74 (±0.34)	3.96 (±0.08)
Wholemeal Semolina	16.28 (±0.09)	1.25 (±0.03)	17.83 (±0.39)	1.17 (±0.06)	56.98 (±0.37)	4.14 (±0.15)
Wheat Germ	17.50 (±0.04)	0.67 (±0.00)	17.89 (±0.07)	1.57 (±0.01)	53.28 (±0.14)	6.29 (±0.04)

Main fatty acids (Area % > 1 % of total FAME) are reported

Mean of, at least, three replicates ± standard error.

* each fatty acid was expressed as percentage on the total fatty acids identified with GC analysis

Table S5. Complete fatty acid profile of Control Pasta (CP) samples during storage at 5°C under modified atmosphere. Each fatty acid was expressed as percentage of the sum of total fatty acids. Statistical analysis results are reported.

	CP										Statistical analysis ANOVA + Tukey's test	
	T0		T15		T35		T55		T75			
	**Mean**	St.dev	**Mean**	St.dev	**Mean**	St.dev	**Mean**	St.dev	**Mean**	St.dev	Significant differences	*p*-value
**SFA (%)**	**20.64**	0.18	**20.54**	0.24	**20.30**	0.19	**20.28**	0.11	**20.15**	0.08		
C14:0	**0.08**	0.00	**0.08**	0.00	**0.07**	0.00	**0.07**	0.00	**0.07**	0.00		
C15:0	**0.11**	0.00	**0.11**	0.01	**0.10**	0.00	**0.10**	0.00	**0.10**	0.00		
C16:0	**18.39**	0.18	**18.28**	0.25	**17.97**	0.31	**17.93**	0.09	**17.75**	0.07		
C17:0	**0.12**	0.01	**0.12**	0.02	**0.11**	0.00	**0.12**	0.01	**0.12**	0.01		
C18:0	**1.46**	0.05	**1.48**	0.04	**1.49**	0.05	**1.49**	0.04	**1.50**	0.03		
C20:0	**0.11**	0.00	**0.11**	0.01	**0.12**	0.00	**0.12**	0.00	**0.12**	0.01		
C21:0	**0.03**	0.00	**0.03**	0.00	**0.02**	0.01	**0.02**	0.01	**0.02**	0.01		
C22:0	**0.18**	0.01	**0.19**	0.01	**0.21**	0.02	**0.22**	0.01	**0.23**	0.00	T55-T0; T75-T0; T55-T15; T75-T15	1.90E-03
C23:0	**0.04**	0.00	**0.04**	0.00	**0.05**	0.00	**0.05**	0.01	**0.06**	0.01		
C24:0	**0.12**	0.01	**0.12**	0.01	**0.15**	0.03	**0.16**	0.01	**0.17**	0.01	T55-T0; T75-T0; T55-T15; T75-T15	3.49E-03
**MUFA (%)**	**15.75**	0.12	**15.78**	0.16	**15.89**	0.31	**15.86**	0.27	**16.04**	0.17		
C16:1 n9	**0.09**	0.00	**0.08**	0.00	**0.09**	0.01	**0.08**	0.00	**0.08**	0.00		
C16:1 n7	**0.14**	0.00	**0.14**	0.01	**0.13**	0.01	**0.13**	0.01	**0.13**	0.00		
C17:1 n8	**0.06**	0.00	**0.05**	0.00	**0.05**	0.00	**0.06**	0.01	**0.06**	0.01		
C18:1 n9	**13.91**	0.14	**13.95**	0.19	**14.05**	0.28	**14.02**	0.29	**14.15**	0.19		
C18:1 n7	**1.02**	0.01	**1.02**	0.01	**1.02**	0.03	**1.01**	0.02	**1.03**	0.02		
C18:1 n5	**0.02**	0.02	**0.01**	0.02	**0.01**	0.01	**0.00**	0.00	**0.00**	0.00		
C20:1 n11	**0.03**	0.00	**0.03**	0.00	**0.03**	0.01	**0.03**	0.01	**0.03**	0.00		
C20:1 n9	**0.41**	0.00	**0.41**	0.01	**0.43**	0.01	**0.43**	0.00	**0.46**	0.02	T75-T0; T75-T15	1.80E-03
C22:1 n9	**0.04**	0.01	**0.04**	0.00	**0.05**	0.00	**0.05**	0.01	**0.05**	0.01		
C24:1 n9	**0.04**	0.00	**0.04**	0.00	**0.05**	0.01	**0.05**	0.01	**0.05**	0.01		
**PUFA (%)**	**62.49**	0.17	**62.56**	0.25	**62.61**	0.13	**62.63**	0.31	**62.56**	0.24		

**n6**	**58.79**	0.21	**58.85**	0.26	**58.89**	0.13	**58.99**	0.36	**58.89**	0.24	
C18:2 n6	**58.70**	0.20	**58.76**	0.23	**58.80**	0.11	**58.90**	0.34	**58.80**	0.22	
C20:2 n6	**0.09**	0.03	**0.09**	0.03	**0.09**	0.02	**0.09**	0.02	**0.09**	0.02	
**n3**	**3.69**	0.10	**3.71**	0.07	**3.70**	0.08	**3.63**	0.05	**3.66**	0.05	
C16:4 n3	**0.06**	0.06	**0.07**	0.04	**0.08**	0.06	**0.02**	0.01	**0.02**	0.01	
C18:3 n3	**3.62**	0.06	**3.62**	0.06	**3.62**	0.05	**3.61**	0.05	**3.65**	0.05	
C20:3 n3	**0.01**	0.01	**0.01**	0.01	**0.00**	0.01	**0.00**	0.00	**0.00**	0.00	
**other n**											
C18:4 n1	**0.01**	0.01	**0.01**	0.02	**0.01**	0.02	**0.01**	0.01	**0.01**	0.01	
**other FA (%)**											
C18:2 n6 trans	**0.04**	0.00	**0.04**	0.01	**0.03**	0.01	**0.03**	0.01	**0.03**	0.01	
C19:1 n10 trans	**0.05**	0.00	**0.05**	0.00	**0.05**	0.00	**0.04**	0.02	**0.04**	0.01	
Unknown 1	**0.11**	0.01	**0.11**	0.01	**0.15**	0.03	**0.16**	0.01	**0.18**	0.01	T55-T0; T75-T0; T55-T15; T75-T15 2.65E-03
Unknown 2	**0.66**	0.04	**0.66**	0.05	**0.69**	0.02	**0.71**	0.04	**0.71**	0.02	
Unknown 3	**0.26**	0.01	**0.26**	0.02	**0.27**	0.00	**0.28**	0.02	**0.28**	0.02	
PUFA/SFA	**3.03**	0.03	**3.05**	0.05	**3.08**	0.02	**3.09**	0.03	**3.11**	0.02	
MUFA/SFA	**0.76**	0.01	**0.77**	0.01	**0.78**	0.02	**0.78**	0.01	**0.80**	0.01	
n3/n6	**0.06**	0.00	**0.06**	0.00	**0.06**	0.00	**0.06**	0.00	**0.06**	0.00	

SFA = Saturated Fatty Acid

MUFA = Monounsaturated Fatty Acid

PUFA = Polyunsaturated Fatty Acid

Table S6. Complete fatty acid profile of Raw Germ Pasta (RGP) samples during storage at 5°C under modified atmosphere. Each fatty acid was expressed as percentage of the sum of total fatty acids. Statistical analysis results are reported.

	**RGP**										**Statistical analysis ANOVA + Tukey's test**	
	**T0**		**T15**		**T35**		**T55**		**T75**			
	**Mean**	St.dev	**Mean**	St.dev	**Mean**	St.dev	**Mean**	St.dev	**Mean**	St.dev	Significant differences	*p*-value
**SFA (%)**	**20.43**	0.30	**20.14**	0.07	**19.91**	0.08	**20.00**	0.18	**20.09**	0.10		
C14:0	**0.09**	0.00	**0.08**	0.00	**0.08**	0.00	**0.08**	0.00	**0.08**	0.00		
C15:0	**0.09**	0.00	**0.09**	0.01	**0.09**	0.00	**0.09**	0.00	**0.09**	0.00		
C16:0	**18.59**	0.28	**18.30**	0.12	**18.00**	0.10	**18.11**	0.20	**18.20**	0.09		
C17:0	**0.08**	0.01	**0.08**	0.01	**0.08**	0.01	**0.09**	0.01	**0.09**	0.01		
C18:0	**1.16**	0.01	**1.18**	0.03	**1.20**	0.01	**1.19**	0.01	**1.19**	0.02		
C20:0	**0.11**	0.01	**0.11**	0.00	**0.12**	0.01	**0.12**	0.01	**0.12**	0.00		
C21:0	**0.01**	0.01	**0.02**	0.00	**0.02**	0.00	**0.02**	0.01	**0.02**	0.00		
C22:0	**0.14**	0.02	**0.15**	0.02	**0.16**	0.01	**0.16**	0.01	**0.16**	0.02		
C23:0	**0.03**	0.00	**0.04**	0.00	**0.04**	0.00	**0.04**	0.00	**0.04**	0.00		
C24:0	**0.09**	0.01	**0.10**	0.01	**0.12**	0.01	**0.11**	0.01	**0.10**	0.01		
**MUFA (%)**	**18.26**	0.13	**18.47**	0.10	**18.51**	0.13	**18.57**	0.23	**18.50**	0.17		
C16:1 n9	**0.04**	0.04	**0.07**	0.00	**0.07**	0.01	**0.07**	0.00	**0.07**	0.00		
C16:1 n7	**0.17**	0.00	**0.16**	0.00	**0.16**	0.00	**0.17**	0.00	**0.17**	0.00		
C17:1 n8	**0.04**	0.00	**0.05**	0.00	**0.05**	0.00	**0.05**	0.01	**0.05**	0.00		
C18:1 n9	**15.97**	0.16	**16.12**	0.09	**16.09**	0.15	**16.17**	0.23	**16.11**	0.16		
C18:1 n7	**1.21**	0.04	**1.20**	0.02	**1.23**	0.02	**1.23**	0.03	**1.22**	0.02		
C18:1 n5	**0.02**	0.01	**0.00**	0.00	**0.01**	0.01	**0.00**	0.01	**0.00**	0.00		
C20:1 n11	**0.03**	0.00	**0.03**	0.00	**0.03**	0.00	**0.03**	0.00	**0.03**	0.00		
C20:1 n9	**0.66**	0.02	**0.69**	0.01	**0.71**	0.01	**0.71**	0.02	**0.70**	0.01		
C22:1 n9	**0.08**	0.01	**0.09**	0.00	**0.09**	0.01	**0.09**	0.01	**0.09**	0.00		
C24:1 n9	**0.05**	0.01	**0.06**	0.01	**0.07**	0.01	**0.07**	0.01	**0.06**	0.00		
**PUFA (%)**	**60.56**	0.33	**60.58**	0.08	**60.70**	0.20	**60.58**	0.34	**60.56**	0.27		
**n6**	**56.02**	0.28	**56.00**	0.14	**56.15**	0.21	**56.01**	0.31	**56.02**	0.30		
C18:2 n6	**55.93**	0.28	**55.91**	0.12	**56.06**	0.19	**55.92**	0.28	**55.93**	0.27		
C20:2 n6	**0.09**	0.02	**0.09**	0.02	**0.08**	0.03	**0.09**	0.03	**0.09**	0.03		

**n3**	**4.53**	0.06	**4.56**	0.08	**4.54**	0.06	**4.56**	0.07	**4.52**	0.04		
C16:4 n3	**0.06**	0.03	**0.06**	0.03	**0.03**	0.01	**0.03**	0.01	**0.04**	0.01		
C18:3 n3	**4.47**	0.08	**4.50**	0.08	**4.51**	0.05	**4.53**	0.07	**4.49**	0.05		
**other n**												
C18:4 n1	**0.02**	0.03	**0.02**	0.03	**0.02**	0.03	**0.02**	0.03	**0.01**	0.02		
**other FA (%)**												
C17:0 iso	**0.04**	0.04	**0.00**	0.00	**0.00**	0.00	**0.00**	0.00	**0.00**	0.00		
C18:2 n6 trans	**0.04**	0.00	**0.04**	0.01	**0.04**	0.01	**0.04**	0.01	**0.04**	0.01		
C19:1 n10 trans	**0.06**	0.01	**0.06**	0.00	**0.07**	0.01	**0.06**	0.01	**0.06**	0.01		
Unknown 1	**0.07**	0.02	**0.09**	0.02	**0.11**	0.01	**0.10**	0.01	**0.09**	0.01		
Unknown 2	**0.46**	0.02	**0.50**	0.02	**0.52**	0.00	**0.51**	0.01	**0.51**	0.01	T15-T0; T35-T0; T55-T0; T75-T0	1.56E-03
Unknown 3	**0.12**	0.01	**0.12**	0.01	**0.14**	0.00	**0.13**	0.01	**0.15**	0.00	T35-T0; T75-T0; T35-T15; T75-T15	1.26E-03
PUFA/SFA	**2.97**	0.06	**3.01**	0.01	**3.05**	0.02	**3.03**	0.04	**3.01**	0.03		
MUFA/SFA	**0.89**	0.01	**0.92**	0.01	**0.93**	0.00	**0.93**	0.01	**0.92**	0.00	T15-T0; T35-T0; T55-T0; T75-T0	2.34E-03
n3/n6	**0.08**	0.00	**0.08**	0.00	**0.08**	0.00	**0.08**	0.00	**0.08**	0.00		

SFA = Saturated Fatty Acid

MUFA = Monounsaturated Fatty Acid

PUFA = Polyunsaturated Fatty Acid

Table S7. Complete fatty acid profile of Sourdough Pasta (SP) samples during storage at 5°C under modified atmosphere. Each fatty acid was expressed as percentage of the sum of total fatty acids. Statistical analysis results are reported.

	SP										Statistical analysis ANOVA + Tukey's test	
	T0		T15		T35		T55		T75			
	**Mean**	St.dev	**Mean**	St.dev	**Mean**	St.dev	**Mean**	St.dev	**Mean**	St.dev	Significant differences	*p*-value
**SFA (%)**	**20.11**	0.05	**20.27**	0.18	**19.96**	0.08	**19.87**	0.11	**19.82**	0.03	T75-T0; T35-T15; T55-T15; T75-T15	2.15E-03
C14:0	**0.08**	0.01	**0.08**	0.00	**0.08**	0.01	**0.08**	0.00	**0.08**	0.00		
C15:0	**0.09**	0.00	**0.09**	0.00	**0.09**	0.00	**0.09**	0.00	**0.09**	0.00		
C16:0	**18.22**	0.09	**18.42**	0.23	**18.03**	0.10	**17.91**	0.11	**17.89**	0.03	T35-T15; T55-T15; T75-T15	2.49E-03
C17:0	**0.09**	0.00	**0.09**	0.01	**0.09**	0.00	**0.09**	0.00	**0.09**	0.01		
C18:0	**1.20**	0.02	**1.19**	0.02	**1.21**	0.01	**1.22**	0.01	**1.21**	0.02		
C20:0	**0.12**	0.01	**0.12**	0.00	**0.12**	0.00	**0.13**	0.00	**0.12**	0.00		
C21:0	**0.03**	0.00	**0.02**	0.01	**0.03**	0.00	**0.02**	0.01	**0.02**	0.01		
C22:0	**0.15**	0.01	**0.14**	0.01	**0.16**	0.01	**0.17**	0.00	**0.17**	0.00	T55-T0; T35-T15; T55-T15; T75-T15	7.91E-04
C23:0	**0.04**	0.00	**0.04**	0.00	**0.04**	0.00	**0.05**	0.00	**0.04**	0.00	T15-T0; T55-T15; T75-T15; T55-T35	1.45E-03
C24:0	**0.10**	0.01	**0.09**	0.01	**0.11**	0.00	**0.12**	0.01	**0.12**	0.01	T55-T0; T75-T0; T35-T15; T55-T15; T75-T15	2.70E-04
**MUFA (%)**	**18.55**	0.19	**18.52**	0.18	**18.72**	0.21	**18.78**	0.16	**18.82**	0.11		
C16:1 n9	**0.07**	0.00	**0.07**	0.00	**0.07**	0.00	**0.07**	0.00	**0.07**	0.00		
C16:1 n7	**0.17**	0.00	**0.17**	0.01	**0.17**	0.01	**0.17**	0.00	**0.17**	0.01		
C17:1 n8	**0.05**	0.00	**0.05**	0.00	**0.05**	0.00	**0.05**	0.00	**0.05**	0.00		
C18:1 n9	**16.11**	0.17	**16.14**	0.18	**16.25**	0.22	**16.29**	0.19	**16.34**	0.16		
C18:1 n7	**1.24**	0.04	**1.23**	0.04	**1.26**	0.06	**1.25**	0.03	**1.25**	0.06		
C18:1 n5	**0.02**	0.01	**0.00**	0.01	**0.00**	0.00	**0.01**	0.00	**0.00**	0.00	T15-T0; T35-T0; T75-T0	3.16E-03
C20:1 n11	**0.03**	0.00	**0.03**	0.00	**0.03**	0.00	**0.03**	0.01	**0.02**	0.02		
C20:1 n9	**0.71**	0.01	**0.69**	0.02	**0.74**	0.01	**0.75**	0.01	**0.75**	0.01	T55-T0; T75-T0; T35-T15; T55-T15; T75-T15	7.76E-04
C22:1 n9	**0.09**	0.01	**0.08**	0.01	**0.09**	0.00	**0.10**	0.00	**0.09**	0.00	T55-T0; T55-T15	1.18E-02
C24:1 n9	**0.06**	0.01	**0.05**	0.00	**0.07**	0.00	**0.07**	0.00	**0.07**	0.01	T35-T15; T55-T15; T75-T15	3.25E-03
**PUFA (%)**	**60.75**	0.16	**60.64**	0.17	**60.70**	0.18	**60.70**	0.29	**60.72**	0.12		
**n6**	**56.16**	0.14	**56.10**	0.14	**56.15**	0.21	**56.14**	0.27	**56.16**	0.07		
C18:2 n6	**56.04**	0.14	**56.00**	0.14	**56.03**	0.20	**56.03**	0.26	**56.04**	0.07		

C20:2 n6	**0.11**	0.01	**0.11**	0.00	**0.11**	0.01	**0.11**	0.00	**0.11**	0.00		
**n3**	**4.59**	0.04	**4.54**	0.03	**4.55**	0.03	**4.56**	0.03	**4.56**	0.06		
C16:4 n3	**0.04**	0.03	**0.03**	0.00	**0.04**	0.01	**0.02**	0.01	**0.02**	0.02		
C18:3 n3	**4.55**	0.01	**4.51**	0.03	**4.51**	0.03	**4.54**	0.02	**4.54**	0.04		
**other FA (%)**												
C18:2 n6 trans	**0.04**	0.01	**0.04**	0.00	**0.04**	0.00	**0.04**	0.01	**0.04**	0.01		
C19:1 n10 trans	**0.06**	0.00	**0.07**	0.00	**0.06**	0.00	**0.06**	0.00	**0.07**	0.01		
Unknown 1	**0.09**	0.01	**0.08**	0.01	**0.11**	0.00	**0.12**	0.01	**0.11**	0.01	T55-T0; T35-T15; T55-T15; T75-T15	2.76E-03
Unknown 2	**0.30**	0.04	**0.29**	0.04	**0.31**	0.04	**0.32**	0.03	**0.32**	0.04		
Unknown 3	**0.10**	0.01	**0.10**	0.02	**0.10**	0.01	**0.10**	0.01	**0.11**	0.01		
PUFA/SFA	**3.02**	0.00	**2.99**	0.03	**3.04**	0.01	**3.05**	0.03	**3.06**	0.01	T55-T15; T75-T15	6.51E-03
MUFA/SFA	**0.92**	0.01	**0.91**	0.02	**0.94**	0.01	**0.95**	0.00	**0.95**	0.01	T55-T15; T75-T15	1.21E-02
n3/n6	**0.08**	0.00	**0.08**	0.00	**0.08**	0.00	**0.08**	0.00	**0.08**	0.00		

SFA = Saturated Fatty Acid

MUFA = Monounsaturated Fatty Acid

PUFA = Polyunsaturated Fatty Aci
